# Explainable machine learning for predicting neurological outcome in hemorrhagic and ischemic stroke patients in critical care

**DOI:** 10.3389/fneur.2024.1385013

**Published:** 2024-06-10

**Authors:** Huawei Wei, Xingshuai Huang, Yixuan Zhang, Guowei Jiang, Ruifeng Ding, Mengqiu Deng, Liangtian Wei, Hongbin Yuan

**Affiliations:** ^1^Department of Anesthesiology, Changzheng Hospital, Second Affiliated Hospital of Naval Medical University, Shanghai, China; ^2^Jiangsu Province Key Laboratory of Anesthesiology, Xuzhou Medical University, Xuzhou, China

**Keywords:** critical care, machine learning, model interpretability, prediction model, stroke

## Abstract

**Aim:**

The objective of this study is to develop accurate machine learning (ML) models for predicting the neurological status at hospital discharge of critically ill patients with hemorrhagic and ischemic stroke and identify the risk factors associated with the neurological outcome of stroke, thereby providing healthcare professionals with enhanced clinical decision-making guidance.

**Materials and methods:**

Data of stroke patients were extracted from the eICU Collaborative Research Database (eICU-CRD) for training and testing sets and the Medical Information Mart for Intensive Care IV (MIMIC IV) database for external validation. Four machine learning models, namely gradient boosting classifier (GBC), logistic regression (LR), multi-layer perceptron (MLP), and random forest (RF), were used for prediction of neurological outcome. Furthermore, shapley additive explanations (SHAP) algorithm was applied to explain models visually.

**Results:**

A total of 1,216 hemorrhagic stroke patients and 954 ischemic stroke patients from eICU-CRD and 921 hemorrhagic stroke patients 902 ischemic stroke patients from MIMIC IV were included in this study. In the hemorrhagic stroke cohort, the LR model achieved the highest area under curve (AUC) of 0.887 in the test cohort, while in the ischemic stroke cohort, the RF model demonstrated the best performance with an AUC of 0.867 in the test cohort. Further analysis of risk factors was conducted using SHAP analysis and the results of this study were converted into an online prediction tool.

**Conclusion:**

ML models are reliable tools for predicting hemorrhagic and ischemic stroke neurological outcome and have the potential to improve critical care of stroke patients. The summarized risk factors obtained from SHAP enable a more nuanced understanding of the reasoning behind prediction outcomes and the optimization of the treatment strategy.

## Introduction

Stroke encompasses a set of conditions characterized by the sudden rupture or occlusion of cerebral blood vessels, ultimately resulting in insufficient blood flow and subsequent damage to brain tissue. Clinically, stroke is broadly classified into two main types—ischemic and hemorrhagic—with the latter comprising intracerebral and subarachnoid hemorrhage forms (1). Stroke affects a staggering one in every four individuals over 25 years of age, rendering it the second most common cause of mortality and third leading cause of disability among adult populations worldwide (2). Approximately 16 million people worldwide suffer from various motor and cognitive impairments as a result of stroke, which are often unavoidable sequelae for stroke patients, and severely affects the mobility and quality of life of stroke victims (3).

Acute stroke patients often enter the intensive care unit (ICU) due to consciousness disorders, cardiopulmonary complications, circulatory instability, or acute thrombolytic therapy (4). Compared with patients admitted to a dedicated neurological ward or stroke unit, those with stroke who are admitted to the ICU exhibit heightened neurological severity, notable impairment of consciousness at a moderate to severe level, often necessitating mechanical ventilation, and encounter an elevated risk of hospital mortality (5, 6). ICU provides complex and resource-intensive treatment for hospitalized patients with severe conditions, but current medical resources are often insufficient to meet the needs of ICU patients, and hospitals face pressure to improve critical care efficiency and reduce costs (7). Early prediction of neurological outcome in critically ill stroke patients can provide important references for patients and their families, and can also guide clinicians to give the best intervention measures to patients.

In contrast to conventional predictive models that rely on established variables for computation, machine learning (ML) approaches offer the distinct advantage of incorporating a broader range of variables that more comprehensively capture the intricacies and inherent unpredictability of human physiology (8, 9). Consequently, ML has emerged as a promising tool in the medical field, with its capacity to integrate abundant variables, extract nuanced insights, and generalize acquired knowledge to novel cases with remarkable efficiency and precision (10, 11). Furthermore, interpretable machine learning is increasingly being applied in clinical research, demonstrating robust clinical applicability and guiding capabilities (12, 13).

In this work, we aimed to construct ML models for early and effective prediction of neurological outcome at hospital discharge in critically ill patients with hemorrhagic and ischemic stroke, and employed the shapley additive explanations (SHAP) methods to elucidate the underlying reasons and decision-making processes involved within the optimal algorithm.

## Materials and methods

### Study design

**Figure 1:**
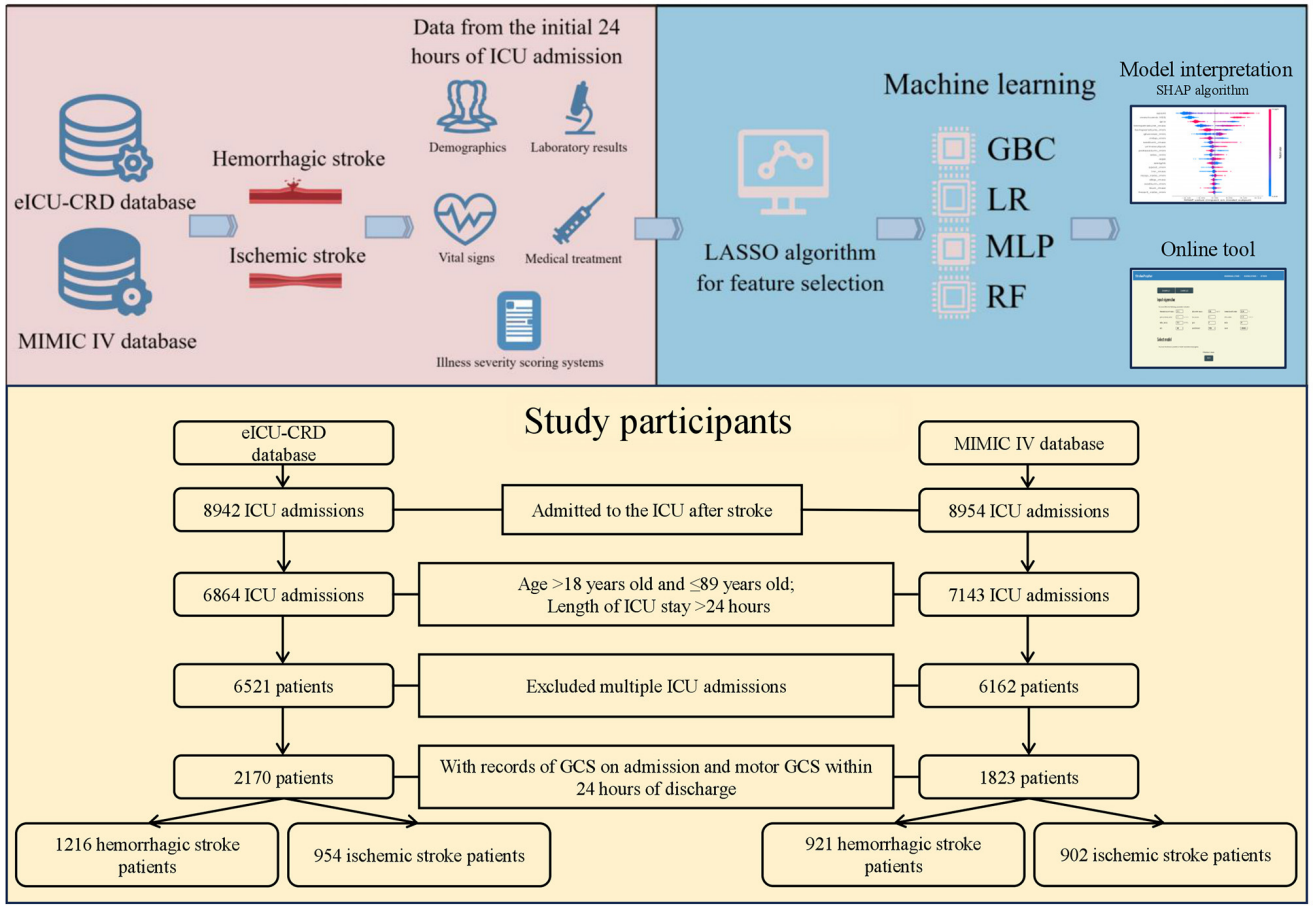
Workflow of the study and flow diagram of study participants inclusion.

The implementation of the study design was shown in Figure 1. The present study was a retrospective modeling study utilizing data from two widely used databases—the eICU Collaborative Research Database (eICU-CRD v2.0) spanning 2014–2015 and the Medical Information Mart for Intensive Care IV (MIMIC IV version 2.2) covering 2008–2019. The author of this study underwent rigorous training, culminating in certification (number 49437998), and was tasked with data extraction following secure access to both databases.

### Participants

In this study, patients diagnosed with stroke according to the ninth and 10th revisions of the international classification of diseases were included (Table 1). These patients were then stratified into hemorrhagic and ischemic cohorts for comparative analysis. Inclusion criteria included individuals over 18 years of age but under 89 years of age who had been in the ICU for at least more than 24 h, along with a Glasgow Coma Scale (GCS) score within 24 h of admission and a documented motor GCS score within 24 h prior to discharge. It is important to note that in the case of repeat ICU admissions, only data relating to the first ICU admission were retained.

**Table 1:** The international classification of diseases codes of the patients included in the study.

**Stroke subtype**	**ICD code**	**ICD version**	**Description**
Hemorrhagic stroke	430	9	Subarachnoid hemorrhage
	431	9	Intracerebral hemorrhage
	432	9	Other and unspecified intracranial hemorrhage
	I60	10	Subarachnoid hemorrhage
	I61	10	Intracerebral hemorrhage
	I62	10	Other non-traumatic intracranial hemorrhage
Ischemic stroke	433	9	Occlusion and stenosis of precerebral arteries
	434	9	Occlusion of cerebral arteries
	I63	10	Cerebral infarction
	I65	10	Occlusion and stenosis of precerebral arteries, not resulting in cerebral infarction
	I66	10	Occlusion and stenosis of cerebral arteries, not resulting in cerebral infarction

### Variables extraction and outcome

In this study, detailed demographic data were collected on age, gender, race, weight, height, and body mass index (BMI). The maximum, minimum and mean values of vital signs during the initial 24 h of ICU admission were extracted, encompassing heart rate (HR), systolic blood pressure (SBP), diastolic blood pressure (DBP), mean blood pressure (MBP), temperature, respiratory rate (RR), and oxygen saturation (SpO_2_). Laboratory parameters obtained within the first 24 h of admission were also extracted. For certain parameters with multiple measurements, both the maximum and minimum values were evaluated. Moreover, the medical interventions employed during the 1st day of admission, such as mechanical ventilation and renal replacement therapy, along with the illness severity scoring systems, namely Charlson comorbidity index, GCS, acute physiology score III (APS III), and sequential organ failure assessment (SOFA), were recorded. Table 2 summarized the variables extracted.

**Table 2:** Clinical features overview.

**Categories**	**Features**
Demographics	Age, gender, race, weight, height, and BMI
Vital signs	Heart rate, systolic blood pressure, diastolic blood pressure, mean blood pressure, temperature, respiratory rate, oxygen saturation, and urineoutput
Laboratory results	Anion gap, bicarbonate, creatinine, chloride, glucose, hematocrit, hemoglobin, lactate, platelet, potassium, ptt, inr, pt, sodium, bun, wbc, and calcium
Medical treatment	Mechanical ventilation, renal replacement therapy, and vasopressor
Illness severity scoring systems	Charlson comorbidity index, Glasgow Coma Scale, Acute Physiology Score III, and Sequential Organ Failure Assessment

BMI, body mass index; PTT, partial thromboplastin time; INR, international normalized ratio; PT, prothrombin time; BUN, blood urea nitrogen; WBC, white blood cell.

The objective of this study was to investigate neurological status at the time of hospital discharge. In the stroke population, the National Institute of Health stroke scale (NIHSS) is a widely accepted metric to determine neurological outcomes. However, since NIHSS was not recorded in eICU-CRD or MIMIC IV, we adopted a surrogate neurological outcome marker based on the motor subscore of the Glasgow Coma Score (mGCS) at discharge. The mGCS score was stratified into two categories, favorable (mGCS score of 6) and unfavorable (mGCS score <5).

### Data preprocessing

In our data preprocessing approach, variables with missing values exceeding 40% were identified as unreliable and thus removed from the dataset to ensure data integrity. Outliers were then detected using the Interquartile Range (IQR) method, computed as the difference between the 75th and 25th percentiles (Q3 and Q1, respectively). Data points falling outside the range of Q1 - 1.5 ^*^ IQR or Q3 + 1.5 ^*^ IQR were flagged as outliers and subsequently eliminated based on statistical conventions. Finally, the multiple imputation method was employed for the imputation of missing numerical values. Renowned for its robustness and capacity to handle intricate datasets, this algorithm efficiently imputes missing values while preserving the inherent data structure.

### Model development

In order to prevent overfitting and simplify the model, we simplified the feature data for each outcome, thereby enabling the models to identify underlying patterns in the data and enhance their generalization capability. To achieve this goal, we utilized the least absolute shrinkage and selection operator (LASSO), a machine learning algorithm, and selected the optimal regularization coefficient lambda through a cross-validation process. Specifically, we opted 10-fold for cross-validation to determine the regularization parameter λ (penalty parameter) in the LASSO algorithm. This parameter facilitates variable selection and shrinkage, enabling the compression of some non-essential variables to zero once λ surpasses a certain threshold, thereby excluding them from the model. Through the computation of performance metrics, such as mean squared error, across various λ values during the cross-validation process, we identified the λ value corresponding to the minimum mean squared error as the final regularization parameter. At this λ value, the non-zero coefficients denote the selected significant features. Our study involved four machine learning models, namely Gradient Boosting Classifier (GBC), Logistic Regression (LR), Multi-Layer Perceptron (MLP), and Random Forest (RF). They are all classification models in supervised learning, with GBC being an ensemble learning model, MLP being a deep learning model, while LR and RF are traditional machine learning models. To optimize model performance, we performed hyperparameter tuning with pre-set hyperparameters (Supplementary Tables 1, 2). The hyperparameter tuning was carried out using 10-fold cross-validation during the training set loop.

### External validation

We conducted external validation using the MIMIC IV dataset, ensuring consistency in patient inclusion criteria and data processing methods with those described for the eICU patient data. Additionally, we ensured that the clinical indicators analyzed in the MIMIC database maintained consistent units with those in the eICU patient data to ensure the accuracy of validation.

### Statistical analysis

We evaluated the predictive performance of our model by measuring several common performance metrics, including accuracy, positive predictive value (PPV), negative predictive value (NPV), sensitivity, specificity, F-measure (F1), and area under the curve (AUC). To determine statistical significance, we used a threshold of *P* < 0.05 and applied Two-tailed Student's *t*-tests or Mann-Whitney *U*-tests for continuous variables, as well as Chi-squared or Fisher's exact tests for categorical variables.

## Results

### Participants

A total of 1,216 hemorrhagic stroke patients and 954 ischemic stroke patients from the eICU-CRD dataset, as well as 921 hemorrhagic stroke patients and 902 ischemic stroke patients from the MIMIC IV dataset, were included in this study. The comparison of baseline features is presented in Table 3, while Supplementary Table 3 provides a summary of specific information for all patients.

**Table 3:** Baseline characteristics of included patients.

	**Hemorrhagic stroke**			**Ischemic stroke**		
	**eICU-CRD**	**MIMIC IV**	***P*** **-value**	**eICU-CRD**	**MIMIC IV**	***P*** **-value**
	**(*****n*** = **1,216)**	**(*****n*** = **921)**		**(*****n*** = **954)**	**(*****n*** = **902)**	
**Demographic**						
Age	64.47 (15.53)	65.63 (15.04)	0.0839	66.38 (14.16)	67.75 (14.02)	0.037
Gender			0.00104			0.00682
Female	656 (53.95 %)	430 (46.69 %)		502 (52.62 %)	417 (46.23 %)	
Male	560 (46.05 %)	491 (53.31 %)		452 (47.38 %)	485 (53.77 %)	
Race			<0.001			<0.001
Asian	17 (1.40 %)	46 (4.99 %)		6 (0.63 %)	23 (2.55 %)	
Black	212 (17.43 %)	90 (9.77 %)		110 (11.53 %)	89 (9.87 %)	
Hispanic	23 (1.89 %)	40 (4.34 %)		11 (1.15 %)	25 (2.77 %)	
Other/unknown	74 (6.09 %)	233 (25.30 %)		35 (3.67 %)	207 (22.95 %)	
White	890 (73.19 %)	512 (55.59 %)		792 (83.02 %)	558 (61.86 %)	
Weight	82.61 (22.55)	80.01 (22.49)	0.00896	83.88 (23.25)	82.31 (23.30)	0.149
**Severity scores on admission**						
Charlson comorbidity index	3.37 (2.48)	5.29 (2.77)	<0.001	3.76 (2.35)	6.11 (2.90)	<0.001
GCS	11.43 (4.37)	12.40 (3.84)	<0.001	12.34 (3.61)	13.03 (3.36)	<0.001
APSIII	46.29 (26.13)	43.49 (23.86)	0.0113	43.15 (24.15)	44.07 (23.93)	0.422
SOFA	3.57 (2.76)	4.21 (3.65)	<0.001	3.28 (2.88)	4.30 (3.81)	<0.001
**First day treatment**						
Vasopressor	62 (5.10 %)	186 (20.20 %)	<0.001	73 (7.65 %)	197 (21.84 %)	<0.001
Renal replacement therapy	13 (1.07 %)	24 (2.61 %)	0.0114	13 (1.36 %)	23 (2.55 %)	0.092
Mechanical ventilation	445 (36.60 %)	421 (45.71 %)	<0.001	277 (29.04 %)	303 (33.59 %)	0.191
Hospital length of stay, day	8.78 (8.74)	8.57 (9.72)	0.622	7.50 (11.59)	9.30 (11.46)	<0.001
ICU length of stay, day	5.86 (6.39)	7.61 (8.06)	<0.001	4.44 (5.72)	7.90 (9.86)	<0.001
**Neurological outcome**						
Favorable	809 (66.53 %)	454 (49.29 %)	<0.001	711 (74.53 %)	540 (59.87 %)	<0.001
Unfavorable	407 (33.47 %)	467 (50.71 %)		243 (25.47 %)	362 (40.13 %)	

Data are n (%) or mean (SD).

GCS, Glasgow Coma Scale; APSIII, Acute Physiology Score III; SOFA, Sequential Organ Failure Assessment.

In cohort extracted from the eICU-CRD, 33.47% (*n* = 407) of hemorrhagic stroke patients had unfavorable neurological outcome at discharge, while 25.47% (*n* = 243) of ischemic stroke patients had unfavorable neurological outcome at discharge. The proportion of unfavorable neurological outcome for hemorrhagic and ischemic stroke patients from MIMIC IV was 50.71% (*n* = 467) and 40.13% (*n* = 362), respectively. Supplementary Table 4 showed the specific information on hemorrhagic and ischemic stroke patients across training and testing sets.

### Feature selection

**Figure 2:**
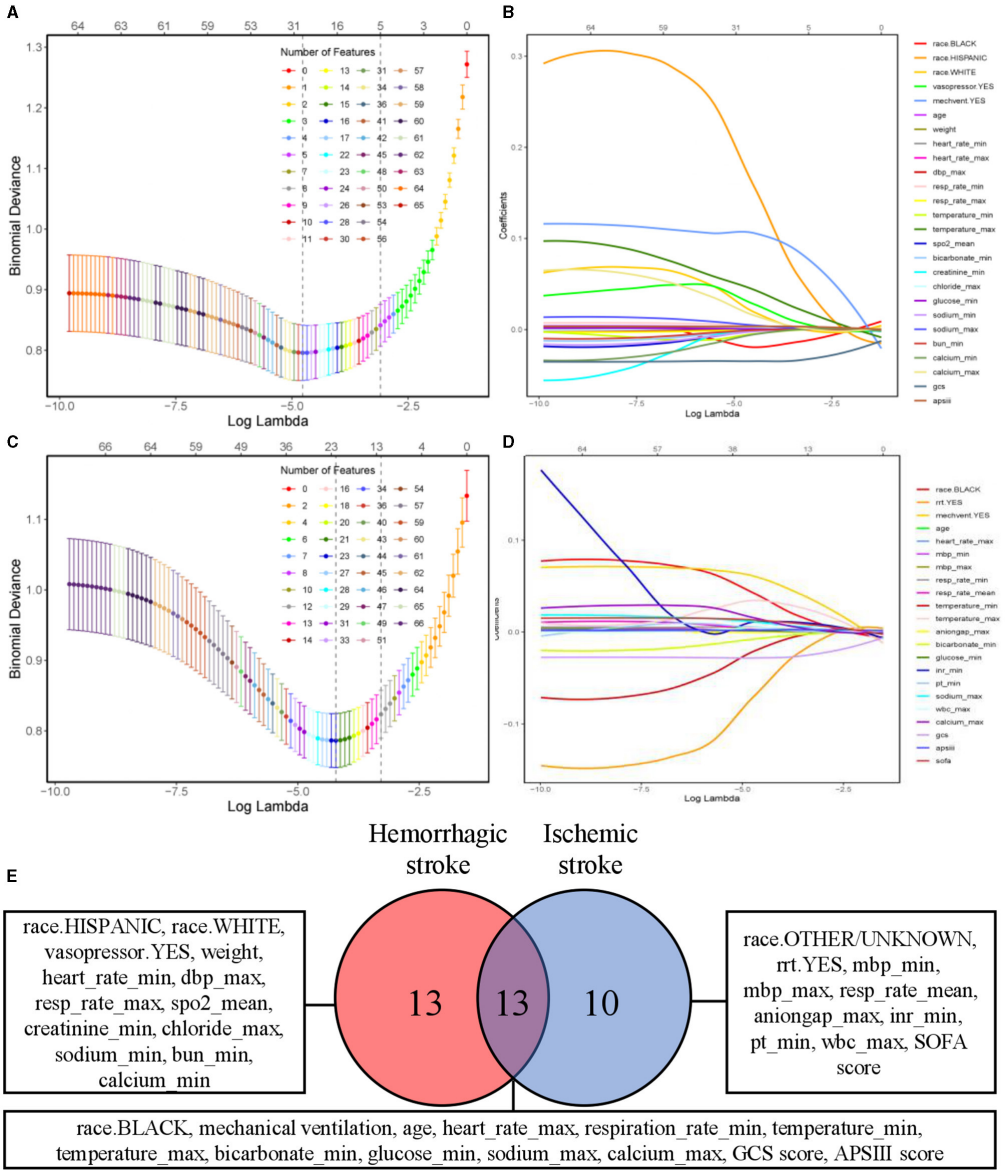
Feature selection using LASSO algorithm. Tuning parameter λ selection by 10-fold cross-validation with minimum criteria. The binomial deviance is plotted on the y-axis against the logarithm of λ on the x-axis. Vertical dotted lines are drawn at the optimal value of λ, which corresponds to the point where the model achieves the best fit to the data. **(A, C)** Depicting partial likelihood deviance of the LASSO regression for neurological outcome of patients with hemorrhagic and ischemic stroke, respectively. **(B, D)** Coefficient profile of the clinical features associated with neurological outcome of patients with hemorrhagic stroke and ischemic stroke. **(E)** Venn diagram of selected features associated with neurological outcome in hemorrhagic and ischemic stroke. LASSO, least absolute shrinkage and selection operator.

The results of feature selection based on the LASSO algorithm was shown in Figures 2A–D. The optimal regularization coefficient lambda for each clinical outcome was selected through a cross-validation process. In hemorrhagic cohort, the optimal lambda value for predicting neurological outcome was 0.01023367 and 0.01618563 in ischemic cohort. Figure 2E showed a Venn diagram of the features selected to predict neurological outcome in hemorrhagic and ischemic stroke. Upon application of the LASSO algorithm, a total of 26 and 23 features were discerned to be associated with neurological outcomes in patients diagnosed with hemorrhagic stroke and ischemic stroke, respectively. Encouragingly, it was observed that 13 features exhibited shared significance across both stroke types, emphasizing potential converging mechanisms influencing neurological outcomes.

### Model performance

**Figure 3:**
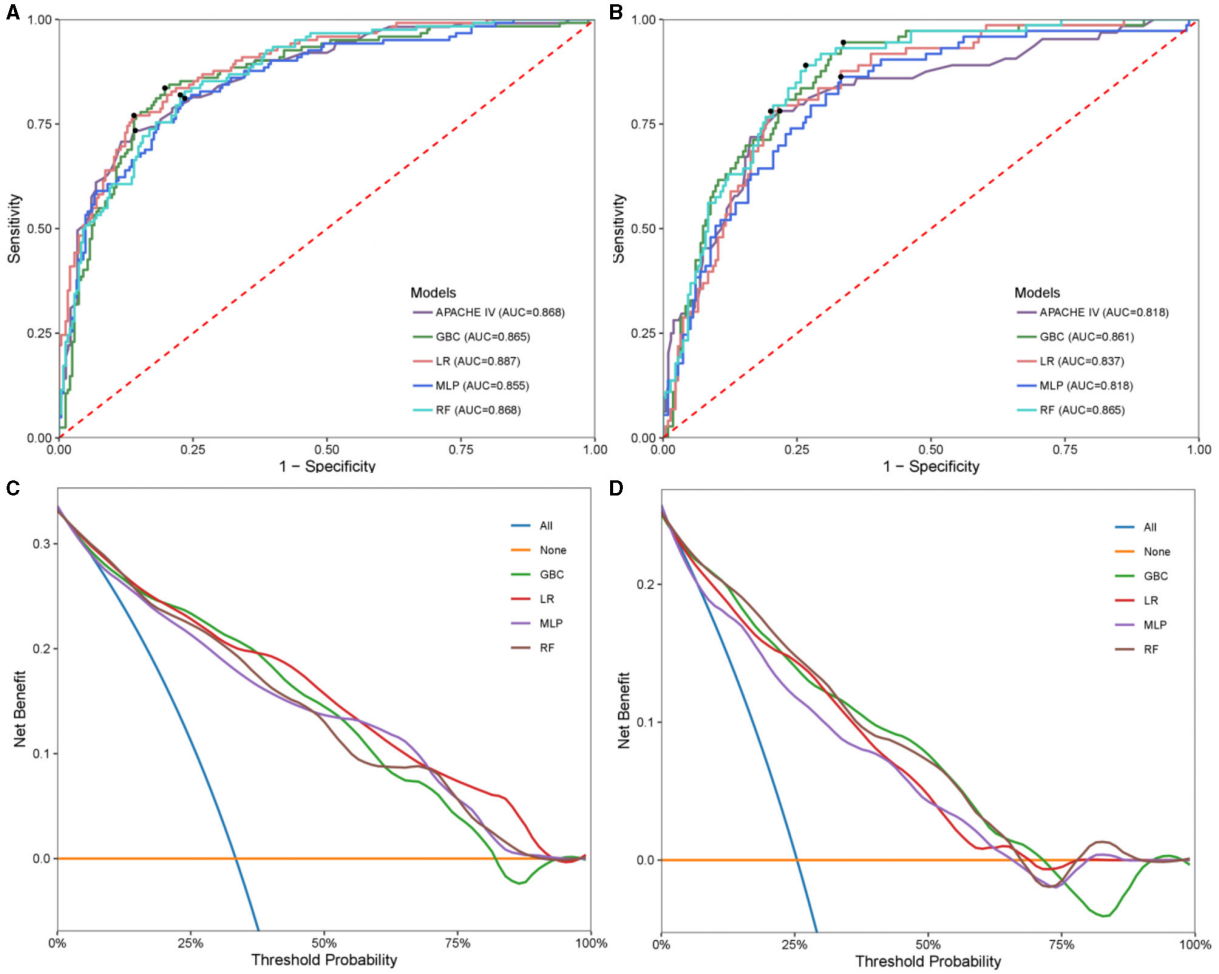
Model performance in hemorrhagic stroke test cohort and ischemic stroke test cohort. Receiver operating characteristic (ROC) analysis of GBC, LR, MLP, RF models, and APACHE reference. **(A)** Hemorrhagic stroke; **(B)** Ischemic stroke. Decision curve analysis (DCA) curves of four machine learning models. **(C)** Hemorrhagic stroke; **(D)** Ischemic stroke. GBC, gradient boosting classifier; LR, logistic regression; MLP, multi-layer perceptron; RF, random forest; APACHE, acute physiology and chronic health evaluation.

Four models, GBC, LR, MLP, and RF were generated to predict neurological outcome in the extracted cohort using the selected features. Acute physiology and chronic health evaluation IV (APACHE IV), a wildly used method for evaluating critically ill patients, was assessed to compared with the generated models in testing set. A set of detailed performance metrics for various machine learning models was presented in Table 4. Figures 3A, C depicted the predictive performances of the four models and APACHE reference in terms of AUC curve and decision curve analysis (DCA) curve. Among the four models, LR model showed the highest accuracy (0.83), PPV (0.734), specificity (0.86), F1 (0.752) and AUC (0.887). The performance of the optimal model was improved compared to APACHE reference. According to the DCA curves of the four predictive models, the net benefit for LR model was larger over the range of the other models.

**Table 4:** Model performance summary of all models in hemorrhagic stroke test cohort.

**Model**	**Accuracy**	**PPV**	**NPV**	**Sensitivity**	**Specificity**	**F1**	**AUC**
GBC	0.814	0.68	0.907	0.836	0.802	0.750	0.865
LR	0.830	0.734	0.882	0.770	0.860	0.752	0.887
MLP	0.781	0.635	0.890	0.811	0.765	0.712	0.855
RF	0.789	0.645	0.895	0.820	0.774	0.722	0.868
APACHE reference	0.817	0.716	0.869	0.735	0.858	0.725	0.868

GBC, gradient boosting classifier; LR, logistic regression; MLP, multi-layer perceptron; RF, random forest; APACHE, acute physiology and chronic health evaluation; PPV, positive predictive value; NPV, negative predictive value; F1, F-measure; AUC, area under curve.

In the same way, the four models were generated to predict neurological outcome in ischemic stroke cohort. Figures 3B, D exhibited the discrimination performance of these models via AUC and DCA curves in the testing set. The predictive performance of each model was presented in Table 5. Of the four models, RF model had the best predictive performance (AUC = 0.867). Besides, RF model had the highest F1 (0.66). In addition, GBC model had the highest NPV (0.973) and sensitivity (0.945) and LR model had the highest accuracy (0.794), PPV (0.57) and specificity (0.799).

**Table 5:** Model performance summary of all models in ischemic stroke test cohort.

**Model**	**Accuracy**	**PPV**	**NPV**	**Sensitivity**	**Specificity**	**F1**	**AUC**
GBC	0.735	0.489	0.973	0.945	0.664	0.645	0.861
LR	0.794	0.570	0.914	0.781	0.799	0.659	0.837
MLP	0.718	0.470	0.935	0.863	0.668	0.609	0.818
RF	0.763	0.520	0.956	0.904	0.715	0.660	0.867
APACHE reference	0.782	0.532	0.919	0.781	0.782	0.633	0.818

GBC, gradient boosting classifier; LR, logistic regression; MLP, multi-layer perceptron; RF, random forest; PPV, positive predictive value; NPV, negative predictive value; F1, F-measure; AUC, area under curve.

### Model interpretation

**Figure 4:**
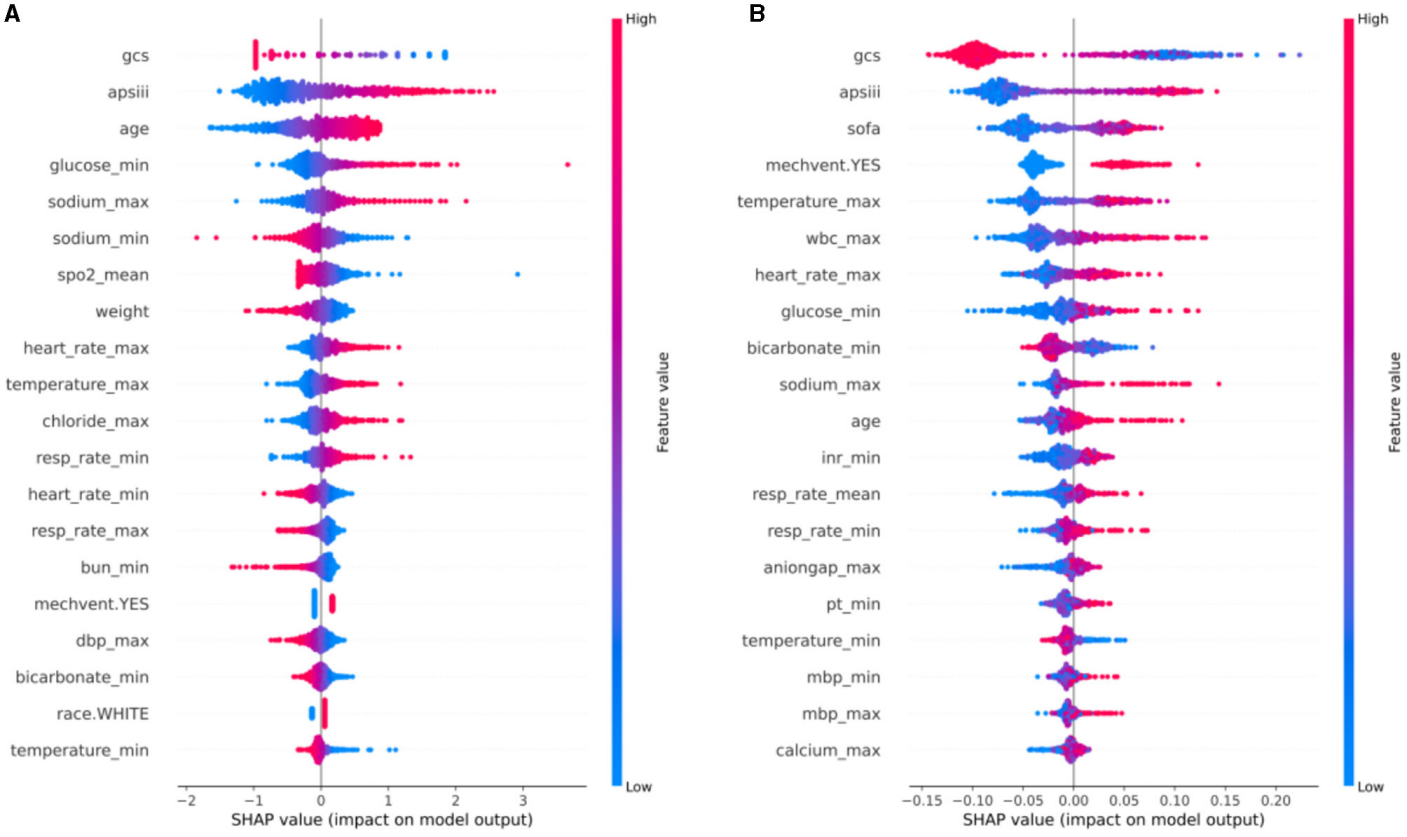
SHAP analysis of the optimal model for neurological outcome in hemorrhagic stroke cohort **(A)** and ischemic stroke cohort **(B)**. SHAP, Shapley additive explanations.

In order to comprehensively elucidate the effect of various clinical features on the neurological outcome of stroke patients, we employed the SHAP algorithm to determine their overall positive or negative impact on the optimal model output. As shown in Figure 4A, GCS score ranked the first in importance among the features for predicting neurological outcome in hemorrhagic stroke cohort, followed by APS III score, age, glucose_min, and sodium_max. Figure 4B showed that GCS score had the most potent predictive power in predicting neurological outcome in ischemic stroke patients, followed by APS III score, SOFA score, mechanical ventilation and temperature_max. To offer a comprehensive overview of feature importance ranking in optimal model construction, Supplementary Figure 1 also provided the summaries of the feature importance ranking for predicting neurological outcome in hemorrhagic and ischemic stroke.

**Figure 5:**
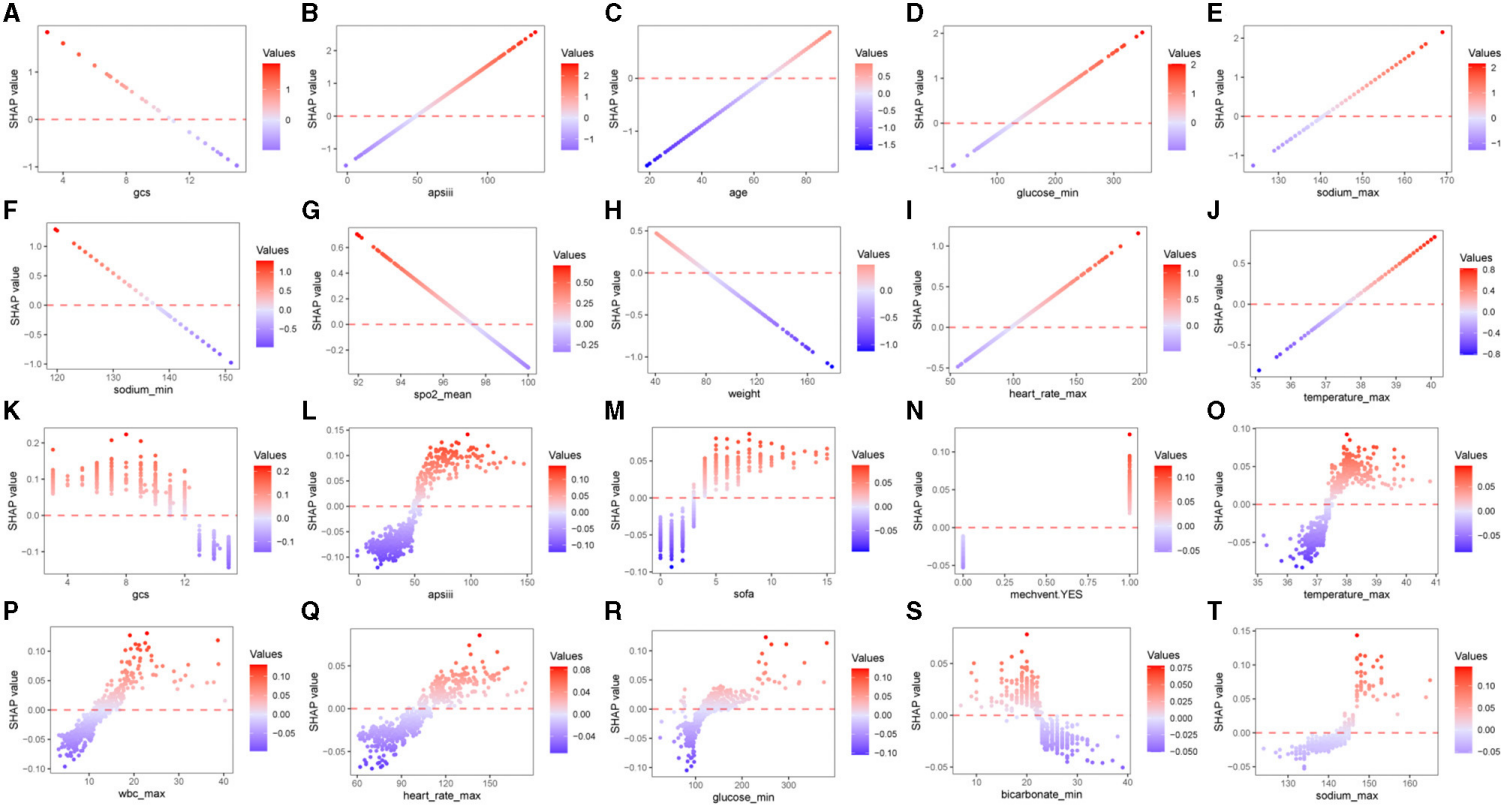
SHAP dependence plots of the top 10 features of predicting neurological outcome in hemorrhagic stroke cohort **(A–J)** and ischemic stroke cohort **(K–T)**. SHAP, Shapley additive explanations; GCS, Glasgow Coma Scale; APS III, Acute Physiology Score III; SpO_2_, Oxygen saturation; SOFA, Sequential Organ Failure Assessment; WBC, white blood cell.

To facilitate a more intuitive understanding of how alterations in individual clinical features impact the model's output, we included SHAP dependence plots that depict the top 10 contributing features for each model in Figure 5, SHAP dependence plots of the remaining features were shown in Supplementary Figures 2, 3. In the case of hemorrhagic stroke, as illustrated in Figures 5A–J, patients with lower GCS score (<11), sodium_min (<137 mmol/L), SpO_2__mean (<97%) and lighter weight (<80 kg) or higher APS III score (>50), glucose_min (>125 mg/dL), sodium_max (>142 mmol/L), heart rate_max (>100 beats/minute), temperature_max (>37.5°C), and order age (>65 years) are more likely to be predicted as having unfavorable neurological outcome. In the ischemic stroke group, the effects of GCS score, APS III score, temperature_max, heart rate_max, glucose_min, and sodium_max on the model's predictions aligned with those observed in the hemorrhagic stroke group. Additionally, ischemic stroke patients with higher SOFA score (>3) and wbc_max (>10^9/*L*^), lower bicarbonate_min (<23 mEq/L) or treated with mechanical ventilation are more prone to be predicted as having unfavorable neurological outcome (Figures 5K–T).

### External validation

Our predictive analyses of the MIMIC IV validation cohorts demonstrated a consistent pattern with that observed in the eICU-CRD cohorts (Supplementary Figure 4). In the validation set, the LR model demonstrated an AUC of 0.836 for predicting neurological outcome in hemorrhagic stroke patients, which represented a decrease of 0.051 compared to the testing set. For the ischemic stroke validation set, the AUC of the RF model was 0.856 for neurological outcome, demonstrating a reduction of 0.011. A comprehensive overview of external validation results is in Supplementary Tables 5, 6.

### Online tool for prediction

**Figure 6:**
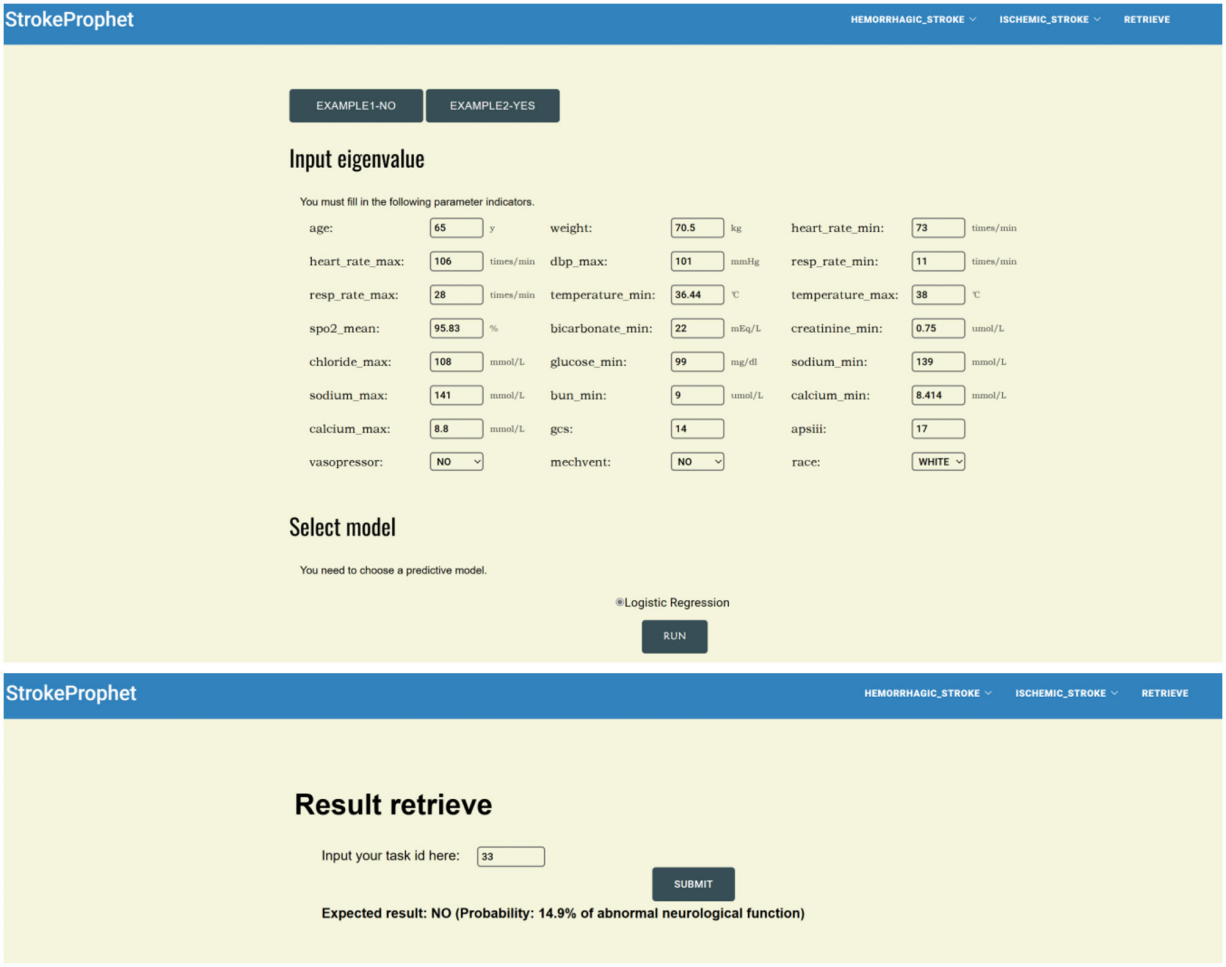
An example of online tool for prediction.

Based on the optimal model for predicting neurological outcome in hemorrhagic and ischemic stroke, along with the relevant clinical variables that it encompasses, we devised an online prediction tool (Figure 6). By selecting the stroke type and entering the relevant clinical data, the user can obtain an automatically generated ID for outcome query. After entering the above ID into the query interface, the user can obtain the neurological outcome prediction result. This prediction tool is accessible at: http://www.strokeprophet.cn.

## Discussion

This retrospective analysis delved into the medical records of stroke patients, utilizing data from the eICU-CRD multicenter database, and effectively validated the findings using the MIMIC IV database. By employing multiple algorithmic techniques and various machine learning models, we successfully identified the features of clinical indicators within the first 24 h of admission that are highly correlated with the neurofunctional state at discharge. Importantly, we integrated the SHAP algorithm and existing literature to provide comprehensive interpretability and inference for these factors, which holds significant value in guiding future prospective research and supporting clinical decision-making in stroke care. Most importantly, the results of this study have been translated into a practical online tool that enables precise prediction of neurologic prognosis in hemorrhagic and ischemic stroke patients using features from the first 24 h in the ICU.

Categorically, stroke can be divided into two primary subtypes: ischemic stroke, accounting for 87% of all cases, and hemorrhagic stroke, comprising intracerebral hemorrhage and subarachnoid hemorrhage, which collectively make up 10 and 3% of stroke occurrences, respectively. Prompt emergency interventions aimed at restoring blood flow prove crucial in improving patient prognosis in the context of ischemic stroke. Conversely, effective management of hemorrhagic stroke necessitates surgical hemostasis and the control of intracranial pressure (14). Existing studies primarily consist of single-center, retrospective observational research aimed at understanding the differences in neurofunctional influencing factors between these two subtypes of stroke (14). However, there is a lack of reported research investigating the neurofunctional prediction and exploration of risk factors specifically in these two stroke patient populations. Our study significantly contributes to filling this gap in knowledge.

Our comprehensive study reveals that within 24 h of hospital admission, ventilator use, age, temperature, GCS score, APS III score, heart rate, blood sodium levels, blood calcium levels, respiratory rate, blood bicarbonate levels, blood glucose levels, and ethnicity all demonstrate noteworthy significance in influencing the prognosis of neurological function for both stroke types. It is imperative to carefully scrutinize and monitor these factors in patients diagnosed with either hemorrhagic or ischemic stroke to facilitate a more accurate assessment of their neurological prognosis.

Recent discoveries indicate that the requirement for mechanical ventilation among stroke patients may exhibit a stronger correlation with the site of brain injury rather than the stroke subtype itself. Consequently, optimizing ventilation strategies assumes a crucial role in enhancing the prognosis of patients within the hemorrhagic stroke cohort (15). In stark contrast, our investigation unveils an intriguing observation: aside from its influence on hemorrhagic stroke outcomes, mechanical ventilator employment also exerts a significant detrimental impact on the prognosis of neurological function in patients suffering from ischemic stroke. Furthermore, our findings identify respiratory rate as a contributing factor affecting the neurological prognosis in both stroke subtypes. These observations may be germane to the development of complicating conditions such as ventilator-associated pneumonia and acute respiratory distress syndrome (16). Therefore, when treating stroke patients, individual circumstances should be carefully considered, mechanical ventilation therapy should be optimized.

Several studies have yielded compelling evidence regarding the influence of hypernatremia and hypernatremia on the neurological prognosis of stroke patients (17, 18). Notably, we have identified a novel association between blood bicarbonate ion levels and the neurological prognosis in both hemorrhagic and ischemic stroke patients, a correlation that has not been previously reported in the literature. The presence of acute hypernatremia and underlying diabetes, recognized risk factors for cerebrovascular disease, further exacerbate the neurological prognosis in stroke patients (19). Additionally, heightened heart rate and elevated body temperature emerge as crucial factors contributing to an unfavorable prognosis among this patient population (20, 21). Our investigation also reveals that advanced age significantly impairs neurological recovery, with older stroke patients exhibiting diminished prognosis relative to their younger counterparts (22). Intriguingly, the observations in elderly mice suggest an enhanced propensity for neutrophil plugging in the ischemic brain microcirculation post-stroke, resulting in compromised blood flow and worsened prognosis (23). Furthermore, dysfunctionality within microglia in aged mouse brains may contribute to the deteriorating neurological prognosis following stroke (24). Similarly, Peng et al.'s report on stroke cohorts from 1990 to 2019 also indicates a relative inadequacy in healthcare for stroke patients across all age groups, underscoring the need for further investigation into the impact of age on mortality trends (25). Disparities in neurological prognosis between racial groups have been noted, with Black patients exhibiting poorer outcomes compared to White patients, potentially linked to stroke subtype (26). Furthermore, Huang et al.'s study also found that race is one of the top 11 most important features for predicting 28-day all-cause in-hospital mortality among hypertensive ischemic or hemorrhagic stroke patients (27). Our study corroborates these findings, highlighting that White patients and Hispanic patients experience inferior neurological prognosis in hemorrhagic stroke, while other ethnic groups fare worse in ischemic stroke. Remarkably, Black patients exhibit poorer prognoses across both stroke subtypes (28). Nevertheless, comprehensive investigations are warranted to validate and elucidate these observations.

Scoring systems play a pivotal role in evaluating the condition and predicting the prognosis of patients, thereby guiding treatment strategies and facilitating informed decision-making. Among these scoring systems, the GCS has widespread application in stroke patients, individuals undergoing open-heart surgery, and those with varying degrees of coma arising from diverse etiologies (29). Our investigation highlights a significant correlation between GCS score and APS III scores and the neurological outcome of patients afflicted by both hemorrhagic and ischemic stroke. Notably, hemorrhagic stroke patients exhibited diminished neurological prognosis when their GCS score was ≤ 11, whereas ischemic stroke patients experienced compromised functional prognosis when their GCS score fell below ≤ 12. However, the underlying mechanisms contributing to these findings necessitate further inquiry. Furthermore, our study reveals that the SOFA score exerts a more pronounced impact on the neurological prognosis of individuals with ischemic stroke relative to the aforementioned scoring metrics (30).

Electrolyte imbalance manifests earlier in individuals suffering from ischemic stroke and holds the potential to serve as a prognostic indicator for neurological outcomes among stroke patients (31). In light of our investigation, it has been established that excessive serum chloride ion concentration (>110 mmol/L) exerts a more pronounced influence on neurological function within the hemorrhagic stroke patient cohort, thereby emphasizing the role of averting hyperchloremia in enhancing neurological outcomes for individuals with hemorrhagic stroke (32). Remarkably, our novel observation reveals that heightened anion gap exhibits a greater impact on the neurological prognosis of ischemic stroke patients compared to ischemic individuals, an unprecedented discovery in current literature. Clinical practitioners should therefore dedicate substantial attention to blood gas analysis results, ensuring prompt correction of electrolyte imbalances while avoiding overcorrection.

Studies in the field of stroke hemodynamics have predominantly concentrated on ischemic stroke due to its higher incidence rate compared to hemorrhagic stroke. Both hypertension and hypotension have detrimental effects on acute ischemic stroke. Generally, antihypertensive therapy is recommended when systolic blood pressure (SBP) exceeds 220 or 180 mmHg in patients eligible for thrombolytic therapy. In the case of acute hemorrhagic stroke, SBP exceeding 140 mmHg is associated with an unfavorable neurological prognosis (33). Notably, our investigation reveals that when monitoring circulatory dynamics in stroke patients, it is crucial to adopt distinct approaches depending on whether the patient has a hemorrhagic or ischemic stroke. For patients with hemorrhagic stroke, the prognostic significance of maximum diastolic blood pressure on neurological function is of greater importance. Conversely, in ischemic patients, both the highest and lowest mean blood pressure levels exert an impact on prognosis. Furthermore, the administration of vasopressors escalates the risk of poor neurological prognosis among individuals with hemorrhagic stroke.

In conjunction with the aforementioned findings, it is imperative to consider several additional factors that warrant separate attention and pertain to patients afflicted with different stroke types. Among individuals with hemorrhagic stroke, maintaining an optimal level of SpO_2_ proves advantageous for prognosis. Notably, accumulating evidence suggests that oxygen therapy is not devoid of risks and should be withheld when SpO_2_ exceeds 90%. Moreover, in patients at risk of hypercapnia, the threshold for oxygen therapy ought to be even lower, specifically ≤ 88%. Once oxygen therapy has been initiated, diligent monitoring of the patient's oxygen saturation and inhaled oxygen concentration becomes paramount in order to maintain SpO_2_ within the targeted range (93–96%) and avert the detrimental consequences of hypercapnia (34). Furthermore, it is worth noting that hemorrhagic stroke patients with lower body weight exhibit an inferior neurological prognosis (35), while diminished levels of blood urea nitrogen are likewise associated with an unfavorable neurological outcome (36).

In the context of individuals suffering from ischemic stroke, heightened emphasis should be placed on monitoring the patient's blood leucocyte count as a means to mitigate infection risk and enhance neurological prognosis (37). Moreover, it is crucial to duly acknowledge the substantial influence exerted by the international normalized ratio (INR) and plasma prothrombin time on ischemic stroke patients, especially following thrombolytic therapy, warranting careful attention (38, 39). These discoveries offer valuable insights into the management of stroke patients necessitating intensive care unit treatment and furnish guidance for improved resource allocation in clinical decision-making.

The final point we wish to emphasize is that machine learning algorithms excel at constructing complex models and making informed decisions when provided with ample and relevant data. In our study, the choice of machine learning models was carefully guided by their unique strengths and documented effectiveness in similar tasks. The GBC model was selected for its exceptional performance in managing high-dimensional data and intricate feature relationships. By leveraging ensemble learning techniques, GBC adeptly captures non-linear associations within the data, making it well-suited for addressing complex classification challenges (40). The RF model was chosen owing to its resilience and efficiency in handling high-dimensional datasets with numerous features (41). Notably, Elsaid et al.'s study on hemorrhagic transformation prediction found that both RFC and GBC models, capable of capturing non-linear interactions among predictor variables, yielded the best predictive performance (AUC: 0.91, 95% CI: 0.85–0.95; AUC: 0.91, 95% CI: 0.86–0.95, respectively) (42). For binary classification tasks, the simplicity, interpretability, and effectiveness of LR made it a prudent choice. Su et al.'s investigation on post-stroke cognitive impairment (PSCI) underscored LR's efficacy in discerning the varying impacts of different factors on cognitive impairment (43). The MLP model was selected for its ability to handle complex non-linear relationships, as demonstrated in Zhou et al.'s study on dementia cognitive footprint recognition (44). MLP emerged as the top-performing model, showcasing satisfactory performance in dementia identification. In our study, the LR model demonstrated the highest AUC of 0.887 in the hemorrhagic stroke cohort, whereas the RF model exhibited superior performance in the ischemic stroke cohort, with an AUC of 0.867. Considering the insightful observations derived from SHAP regarding the importance of variables in predicting each stroke subtype, we analyze and speculate that the primary reason for the differences in optimal models lies in the interactions among predictive factors. Specifically, in hemorrhagic stroke prediction, the most crucial variables predominantly consisted of continuous variables such as age, weight, and glucose. This suggests that LR, with its linear decision boundary, may be better suited to capture the linear relationships among these predictors, thereby achieving higher predictive performance. Conversely, in ischemic stroke prediction, notable variables included the use of mechanical ventilation (mechvent). This intriguing finding suggests that RF, with its capacity to capture complex non-linear relationships, may excel in identifying intricate patterns involving categorical variables like mechvent, which LR may overlook due to its linear nature. These findings lead us to hypothesize that LR's superior performance in hemorrhagic stroke prediction may be attributed to the predominantly linear relationships among predictors, while RF's effectiveness in predicting ischemic stroke-induced damage may stem from its ability to capture non-linear associations. However, further research and validation are warranted to confirm these hypotheses and gain deeper insights into the underlying mechanisms driving model performance discrepancies between stroke subtypes.

The present study has several limitations that warrant consideration. Firstly, our analysis was retrospective in nature, which resulted in the exclusion of certain variables with high missing rates but potentially significant predictive value, such as lactate levels. Secondly, our inability to directly evaluate patients necessitated reliance on diagnostic codes to define our patient cohort, raising the possibility of incorrect associations due to misclassifications. Thirdly, our focus on patients who remained in the ICU for over 24 h and had an mGCS score 24 h before discharge resulted in the exclusion of a sizable number of patients, and this may have introduced some bias into our results. In addition, some important features, namely neuroimaging and electrophysiological examinations, were not included in this study due to the limitations of the database.

## Conclusions

In summary, we employed advanced machine learning techniques to identify and compare the shared and distinct factors that influence hospital discharge outcomes in both hemorrhagic and ischemic stroke. By elucidating these factors, our research will contribute to the advancement of knowledge in the field of stroke, inform medical decision-making, and guide personalized treatment strategies. Our results also demonstrate that machine learning models outperform single standard scoring systems, with the potential to ultimately improve patient outcomes.

## Data availability statement

The original contributions presented in the study are included in the article/Supplementary material, further inquiries can be directed to the corresponding author.

## Ethics statement

Ethical review and approval was not required for the study on human participants in accordance with the local legislation and institutional requirements. Written informed consent from the patients/participants or patients/participants' legal guardian/next of kin was not required to participate in this study in accordance with the national legislation and the institutional requirements.

## Author contributions

HW: Writing—original draft, Writing—review & editing. XH: Writing—original draft, Writing—review & editing. YZ: Writing—original draft, Writing—review & editing. GJ: Writing—original draft, Writing—review & editing. RD: Writing—review & editing. MD: Writing—review & editing. LW: Writing—review & editing. HY: Writing—original draft, Writing—review & editing.

## References

[1] GBD 2016 Neurology Collaborators. Global, regional, and national burden of neurological disorders, 1990-2016: a systematic analysis for the Global Burden of Disease Study 2016. Lancet Neurol. (2019) 18:459–80. 10.1016/S1474-4422(18)30499-X 30879893 PMC6459001

[2] GBD 2019 Stroke Collaborators. Global, regional, and national burden of stroke and its risk factors, 1990-2019: a systematic analysis for the Global Burden of Disease Study 2019. Lancet Neurol. (2021) 20:795–820. 10.1016/S1474-4422(21)00252-0 34487721 PMC8443449

[3] Zhang X Fei N Zhang X Wang Q Fang Z. Machine learning prediction models for postoperative stroke in elderly patients: analyses of the MIMIC database. Front Aging Neurosci. (2022) 14:897611. 10.3389/fnagi.2022.897611 35923545 PMC9341133

[4] Joundi R Smith EE Yu AYX Rashid M Fang J Kapral MK. Age-specific and sex-specific trends in life-sustaining care after acute stroke. J Am Heart Assoc. (2021) 10:e021499. 10.1161/JAHA.121.021499 34514807 PMC8649550

[5] Mayer SA Copeland D Bernardini GL Boden-Albala B Lennihan L Kossoff S. Cost and outcome of mechanical ventilation for life-threatening stroke. Stroke. (2000) 31:2346–53. 10.1161/01.STR.31.10.2346 11022062

[6] van Valburg MK Arbous MS Georgieva M Brealey DA Singer M Geerts BF. Clinical predictors of survival and functional outcome of stroke patients admitted to critical care. Crit Care Med. (2018) 46:1085–92. 10.1097/CCM.0000000000003127 29608513

[7] Diaz JV Riviello ED Papali A Adhikari NKJ Ferreira JC. Global critical care: moving forward in resource-limited settings. Ann Glob Health. (2019) 85:2413. 10.5334/aogh.2413 30741504 PMC7052346

[8] Heo J Yoon JG Park H Kim YD Nam HS Heo JH. Machine learning-based model for prediction of outcomes in acute stroke. Stroke. (2019) 50:1263–5. 10.1161/STROKEAHA.118.024293 30890116

[9] Xu X Qu J Zhang Y Qian X Chen T Liu Y. Development and validation of an MRI-radiomics nomogram for the prognosis of pancreatic ductal adenocarcinoma. Front Oncol. (2023) 13:1074445. 10.3389/fonc.2023.1074445 36910599 PMC9998897

[10] van Os H Ramos LA Hilbert A van Leeuwen M van Walderveen MAA Kruyt ND. Predicting outcome of endovascular treatment for acute ischemic stroke: potential value of machine learning algorithms. Front Neurol. (2018) 9:784. 10.3389/fneur.2018.00784 30319525 PMC6167479

[11] Zhang Y Zhu S Yuan Z Li Q Ding R Bao X. Risk factors and socio-economic burden in pancreatic ductal adenocarcinoma operation: a machine learning based analysis. BMC Cancer. (2020) 20:1161. 10.1186/s12885-020-07626-2 33246424 PMC7694304

[12] Peng S Huang J Liu X Deng J Sun C Tang J. Interpretable machine learning for 28-day all-cause in-hospital mortality prediction in critically ill patients with heart failure combined with hypertension: a retrospective cohort study based on medical information mart for intensive care database-IV and eICU databases. Front Cardiovasc Med. (2022) 9:994359. 10.3389/fcvm.2022.994359 36312291 PMC9597462

[13] Huang J Jin W Duan X Liu X Shu T Fu L. Twenty-eight-day in-hospital mortality prediction for elderly patients with ischemic stroke in the intensive care unit: interpretable machine learning models. Front Public Health. (2022) 10:1086339. 10.3389/fpubh.2022.1086339 36711330 PMC9878123

[14] Salvadori E Papi G Insalata G Rinnoci V Donnini I Martini M. Comparison between ischemic and hemorrhagic strokes in functional outcome at discharge from an intensive rehabilitation hospital. Diagnostics. (2020) 11:38. 10.3390/diagnostics11010038 33379391 PMC7824133

[15] Robba C Bonatti G Battaglini D Rocco PRM Pelosi P. Mechanical ventilation in patients with acute ischaemic stroke: from pathophysiology to clinical practice. Crit Care. (2019) 23:388. 10.1186/s13054-019-2662-8 31791375 PMC6889568

[16] Huang M Gedansky A Hassett CE Price C Fan TH Stephens RS. Pathophysiology of brain injury and neurological outcome in acute respiratory distress syndrome: a scoping review of preclinical to clinical studies. Neurocrit Care. (2021) 35:518–27. 10.1007/s12028-021-01309-x 34297332 PMC8299740

[17] Yuen KCJ Sharf V Smith E Kim M Yuen ASM MacDonald PR. Sodium and water perturbations in patients who had an acute stroke: clinical relevance and management strategies for the neurologist. Stroke Vasc Neurol. (2022) 7:258–66. 10.1136/svn-2021-001230 34969834 PMC9240457

[18] Chi C Patel S Cheung N. Admission sodium levels and hospital outcomes. Intern Med J. (2021) 51:93–8. 10.1111/imj.14777 32043721

[19] Kim S Kim JY Kim ES Park IR Ha EY Chung SM. Early glycaemic variability increases 28-day mortality and prolongs intensive care unit stay in critically ill patients with pneumonia. Ann Med. (2022) 54:2736–43. 10.1080/07853890.2022.2128399 36205625 PMC9553150

[20] Siebert J Gutknecht P Molisz A Trzeciak B Nyka W. Hemodynamic findings in patients with brain stroke. Archiv Med Sci. (2012) 8:371–4. 10.5114/aoms.2012.28567 22662014 PMC3361052

[21] Kallmünzer B Kollmar R. Temperature management in stroke—an unsolved, but important topic. Cerebrovasc Dis. (2011) 31:532–43. 10.1159/000324621 21487217

[22] Roy-O'Reilly M McCullough L. Age and sex are critical factors in ischemic stroke pathology. Endocrinology. (2018) 159:3120–31. 10.1210/en.2018-00465 30010821 PMC6963709

[23] Gullotta G De Feo D Friebel E Semerano A Scotti GM Bergamaschi A. Age-induced alterations of granulopoiesis generate atypical neutrophils that aggravate stroke pathology. Nat Immunol. (2023) 24:925–40. 10.1038/s41590-023-01505-1 37188941

[24] Arbaizar-Rovirosa M Pedragosa J Lozano JJ Casal C Pol A Gallizioli M. Aged lipid-laden microglia display impaired responses to stroke. EMBO Mol Med. (2023) 15:e17175. 10.15252/emmm.202217175 36541061 PMC9906381

[25] Peng S Liu X Cao W Liu Y Liu Y Wang W. Global, regional, and national time trends in mortality for stroke, 1990-2019: an age-period-cohort analysis for the global burden of disease 2019 study and implications for stroke prevention. Int J Cardiol. (2023) 383:117–31. 10.1016/j.ijcard.2023.05.001 37150213

[26] Simmonds K Luo Z Reeves M. Race/ethnic and stroke subtype differences in poststroke functional recovery after acute rehabilitation. Arch Phys Med Rehabil. (2021) 102:1473–81. 10.1016/j.apmr.2021.01.090 33684363

[27] Huang J Chen H Deng J Liu X Shu T Yin C. Interpretable machine learning for predicting 28-day all-cause in-hospital mortality for hypertensive ischemic or hemorrhagic stroke patients in the ICU: a multi-center retrospective cohort study with internal and external cross-validation. Front Neurol. (2023) 14:1185447. 10.3389/fneur.2023.1185447 37614971 PMC10443100

[28] Alter M. Black-white differences in stroke frequency: challenges for research. Neuroepidemiology. (1994) 13:301–7. 10.1159/000110395 7800109

[29] Handschu R Haslbeck M Hartmann A Fellgiebel A Kolominsky-Rabas P Schneider D. Mortality prediction in critical care for acute stroke: severity of illness-score or coma-scale? J Neurol. (2005) 252:1249–54. 10.1007/s00415-005-0853-5 15917980

[30] Qin W Zhang X Yang L Li Y Yang S Li X. Predictive value of the sequential organ failure assessment (SOFA) score for prognosis in patients with severe acute ischemic stroke: a retrospective study. J Int Med Res. (2020) 48:300060520950103. 10.1177/0300060520950103 32865055 PMC7469749

[31] Jhou H-J Chen P-H Yang L-Y Chang S-H Lee C-H. Plasma anion gap and risk of in-hospital mortality in patients with acute ischemic stroke: analysis from the MIMIC-IV database. J Personal Med. (2021) 11:1004. 10.3390/jpm11101004 34683145 PMC8541378

[32] Qureshi A Huang W Hanley DF Hsu CY Martin RH Malhotra K. Early hyperchloremia is independently associated with death or disability in patients with intracerebral hemorrhage. Neurocritical Care. (2022) 37:487–96. 10.1007/s12028-022-01514-2 35513751

[33] Donovan A Flexman A Gelb A. Blood pressure management in stroke. Curr Opin Anaesthesiol. (2012) 25:516–22. 10.1097/ACO.0b013e32835721a5 22895120

[34] Chu D Kim LH Young PJ Zamiri N Almenawer SA Jaeschke R. Mortality and morbidity in acutely ill adults treated with liberal versus conservative oxygen therapy (IOTA): a systematic review and meta-analysis. Lancet. (2018) 391:1693–705. 10.1016/S0140-6736(18)30479-3 29726345

[35] Shiozawa M Kaneko H Itoh H Morita K Okada A Matsuoka S. Association of body mass index with ischemic and hemorrhagic stroke. Nutrients. (2021) 13:72343. 10.3390/nu13072343 34371853 PMC8308685

[36] Peng R Liu K Li W Yuan Y Niu R Zhou L. Blood urea nitrogen, blood urea nitrogen to creatinine ratio and incident stroke: the Dongfeng-Tongji cohort. Atherosclerosis. (2021) 333:1–8. 10.1016/j.atherosclerosis.2021.08.011 34390959

[37] Hu Z-B Zhong Q-Q Lu Z-X Zhu F. Association of platelet-to-white blood cell ratio and platelet-to-neutrophil ratio with the risk of fatal stroke occurrence in middle-aged to older Chinese. BMC Geriatr. (2022) 22:430. 10.1186/s12877-022-03134-z 35581556 PMC9112464

[38] Odén A Fahlén M Hart R. Optimal INR for prevention of stroke and death in atrial fibrillation: a critical appraisal. Thromb Res. (2006) 117:493–9. 10.1016/j.thromres.2004.11.025 16517250

[39] Huang X Moreton FC Kalladka D Cheripelli BK MacIsaac R Tait RC. Coagulation and fibrinolytic activity of tenecteplase and alteplase in acute ischemic stroke. Stroke. (2015) 46:3543–6. 10.1161/STROKEAHA.115.011290 26514192

[40] Ruksakulpiwat S Thongking W Zhou W Benjasirisan C Phianhasin L Schiltz NK. Machine learning-based patient classification system for adults with stroke: a systematic review. Chronic Illn. (2023) 19:26–39. 10.1177/17423953211067435 34903091

[41] Hu J Szymczak S. A review on longitudinal data analysis with random forest. Brief Bioinform. (2023) 24:bbad002. 10.1093/bib/bbad002 36653905 PMC10025446

[42] Elsaid AF Fahmi RM Shehta N Ramadan BM. Machine learning approach for hemorrhagic transformation prediction: capturing predictors' interaction. Front Neurol. (2022) 13:951401. 10.3389/fneur.2022.951401 36504664 PMC9731336

[43] Su W Li H Dang H Han K Liu J Liu T. Predictors of cognitive functions after stroke assessed using the wechsler adult intelligence scale: a retrospective study. J Alzheimers Dis. (2024) 98:109–17. 10.3233/JAD-230840 38363609

[44] Zhou J Liu W Zhou H Lau KK Wong GHY Chan WC. Identifying dementia from cognitive footprints in hospital records among Chinese older adults: a machine-learning study. Lancet Reg Health West Pac. (2024) 46:101060. 10.1016/j.lanwpc.2024.10106 38638410 PMC11025003

