# Supplementary material for: Explainable machine learning for predicting neurological outcome in hemorrhagic and ischemic stroke patients in critical care
Source: Front Neurol. 2024 Jun 10;15:1385013. doi: 10.3389/fneur.2024.1385013 (PMC11194386; doi:10.3389/fneur.2024.1385013)
Supplement: Supplementary file 1 [file Data_Sheet_1.docx]

Supplementary Material


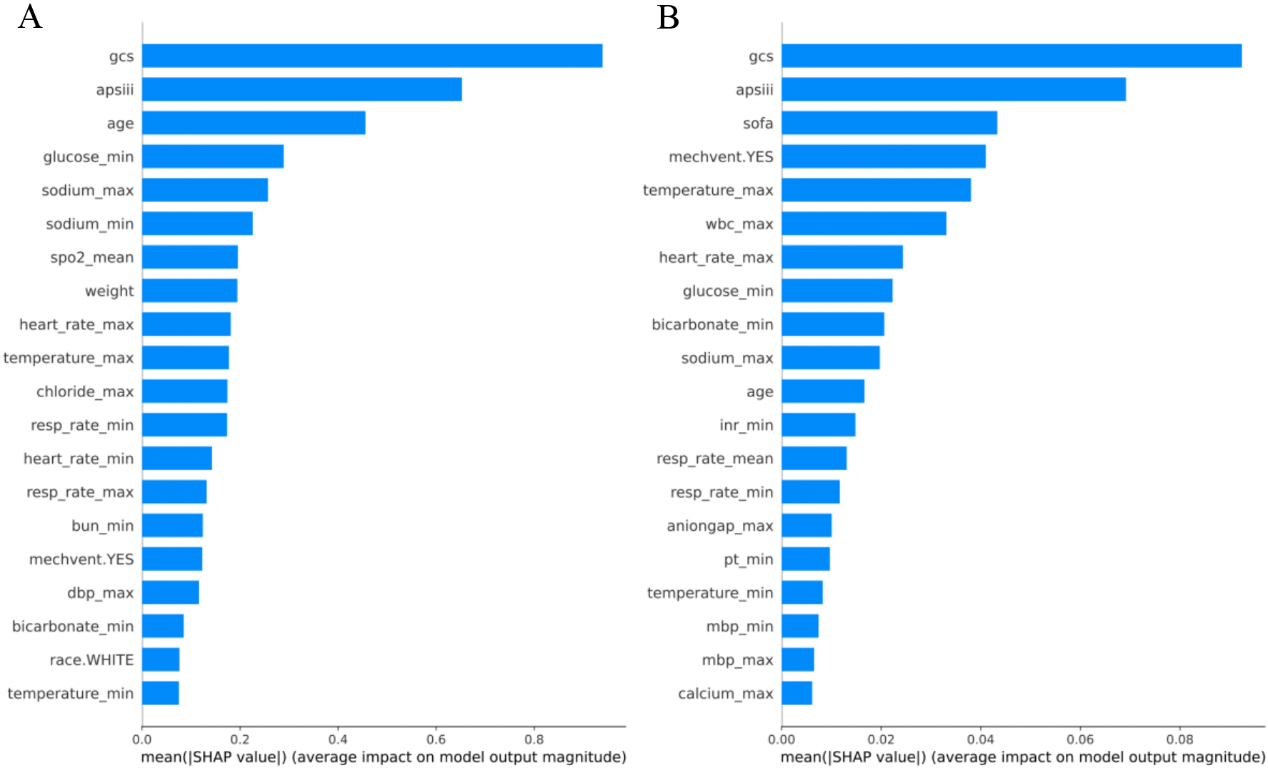


**Supplementary Figure 1.** Bar charts ranking the importance of the top 20 significant features most correlated to neurological outcome in hemorrhagic stroke cohort (A) and ischemic stroke cohort (B).

**
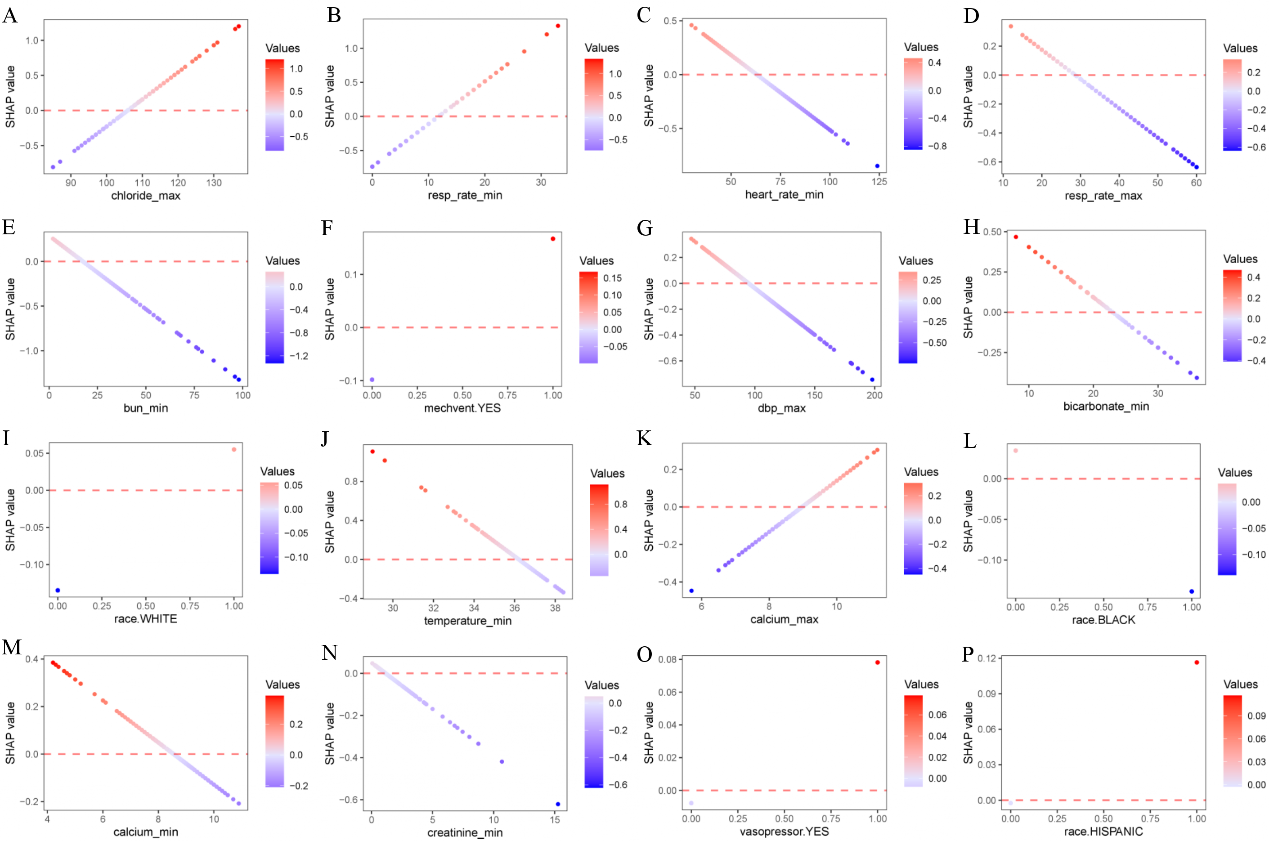
**

**Supplementary Figure 2.** SHAP dependence plots of of the remaining features of predicting neurological outcome in hemorrhagic stroke cohort. (A) chloride_max; (B) respiratory rate_min; (C) heart rate_min; (D) respiratory rate_max; (E) bun_min; (F) mechanical ventilation.YES; (G) dbp_max; (H) bicarbonate_min; (I) race.WHITE; (J) temperature_min; (K) calcium_max; (L) race.BLACK; (M) calcium_min; (N) creatinine_min; (O) vasopressor.YES; (P) race.HISPANIC. SHAP: Shapley additive explanations; bun: blood urea nitrogen; dbp: diastolic blood pressure.


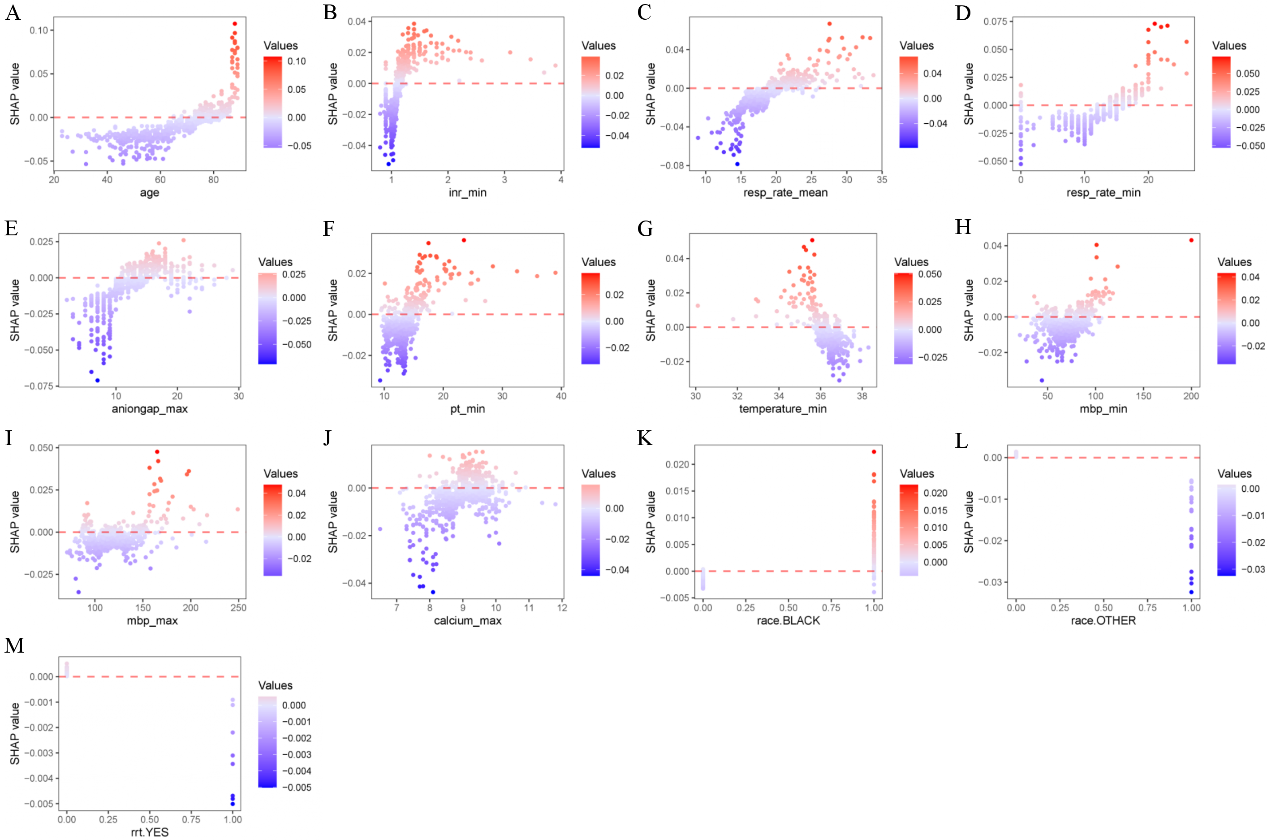
**Supplementary Figure 3.** SHAP dependence plots of of the remaining features of predicting neurological outcome in ischemic stroke cohort. (A) age; (B) inr_min; (C) respiratory rate_mean; (D) respiratory rate_min; (E) aniongap_max; (F) pt_min; (G) temperature_min; (H) mbp_min; (I) mbp_max; (J) calcium_max; (K) race.BLACK; (L) race.OTHER; (M) rrt.YES. SHAP: Shapley additive explanations; inr: international normalized ratio; pt: prothrombin time; mbp: mean blood pressure; rrt: renal replacement therapy.


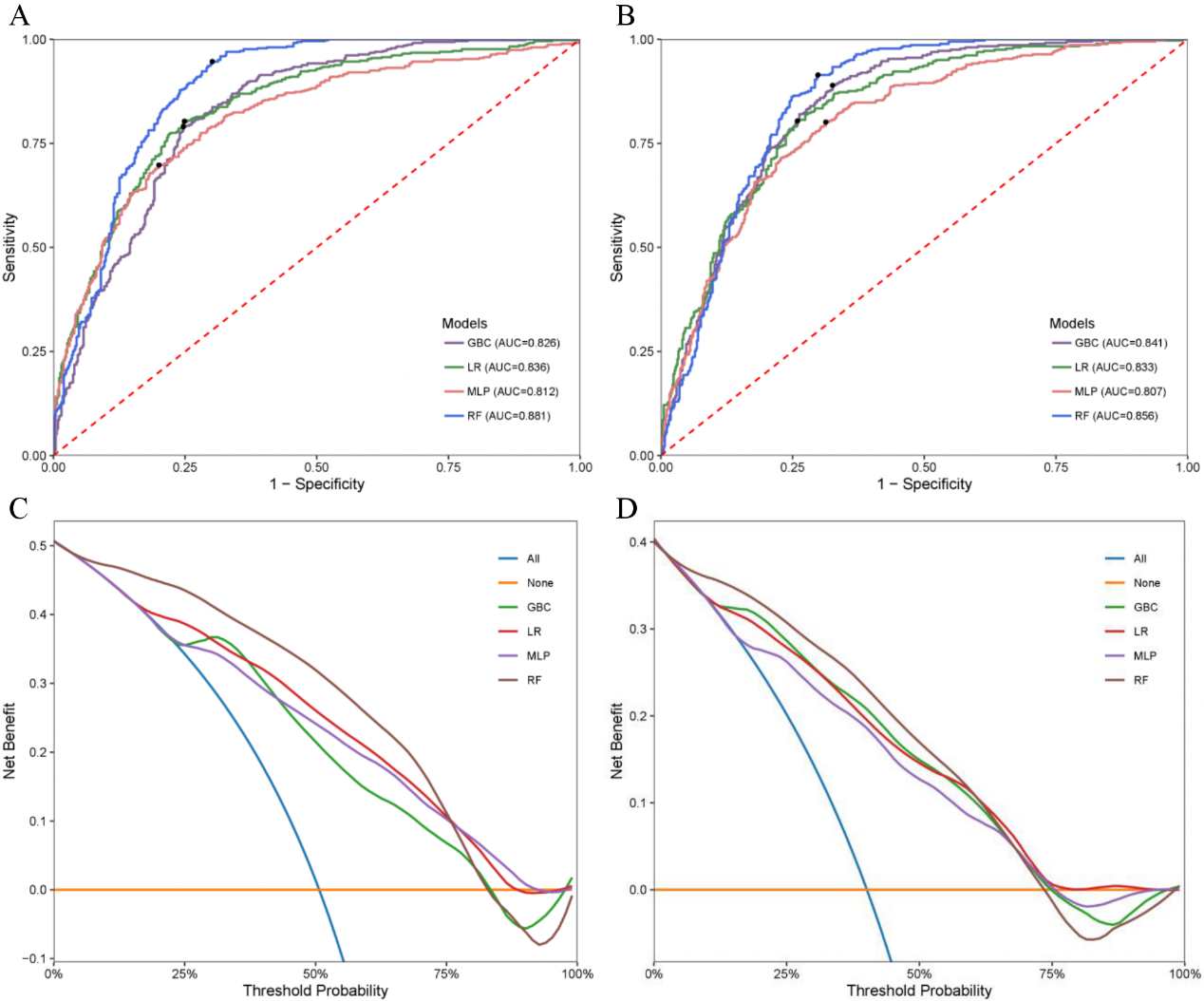


**Supplementary Figure 4.** External validation in the MIMIC IV database. Receiver operating characteristic (ROC) analysis of GBC, LR, MLP and RF models. (A) Hemorrhagic stroke; (B) Ischemic stroke. Decision curve analysis (DCA) curves of four machine learning models. (A) Hemorrhagic stroke; (B) Ischemic stroke. MIMIC IV: Medical Information Mart for Intensive Care IV; GBC: gradient boosting classifier; LR: logistic regression; MLP: multi-layer perceptron; RF: random forest.

**Supplementary Table 1**. Results of hyper-parameter optimization of different machine learning algorithms in hemorrhagic stroke cohort.

| Model | Hyper-parameter space | Best Combination of Hyperparameters |
| --- | --- | --- |
| GBC | {'learning_rate': [1, 0.5, 0.4, 0.3, 0.2, 0.1, 0.01], 'max_depth': [1, 2, 3, 4, 5, 10, 20, 30, 40], 'min_samples_leaf': [0.1, 0.3, 0.5], 'min_samples_split': [0.001, 0.1, 0.3, 0.5, 0.7, 0.9], 'n_estimators': [10, 25, 50, 100, 200, 400, 800]} | {'learning_rate': 0.1, 'max_depth': 1, 'min_samples_leaf': 0.3, 'min_samples_split': 0.1, 'n_estimators': 400} |
| LR | {'C': [0.001, 0.01, 0.1, 1, 10, 100], 'penalty':['l2'], 'solver': ['newton-cg', 'lbfgs', 'liblinear', 'sag', 'saga']} | {'C': 0.1, 'penalty': 'l2', 'solver': 'sag'} |
| MLP | {'activation':['tanh','relu'], 'alpha': [0.1, 0.01, 0.001, 0.0001], 'hidden_layer_sizes':[(50,),(100,)], 'solver':['sgd', 'adam'], 'learning_rate':['constant', 'adaptive']} | {'activation': 'relu', 'alpha': 0.0001, 'hidden_layer_sizes': (50,), 'learning_rate': 'adaptive', 'solver': 'sgd'} |
| RF | {'max_depth': [1, 2, 3, 4, 5, 8, 10, 20, 30, 40, None], 'max_features': ['log2', 'sqrt'], 'min_samples_leaf': [1,2,4], 'min_samples_split': [2,5,10,15], 'n_estimators':[10, 25, 50, 100, 200, 400, 800]} | {'max_depth': 4, 'max_features': 'log2', 'min_samples_leaf': 2, 'min_samples_split': 2, 'n_estimators': 50} |

GBC: gradient boosting classifier; LR: logistic regression; MLP: multi-layer perceptron; RF: random forest.

**Supplementary Table 2.** Results of hyper-parameter optimization of different machine learning algorithms in ischemic stroke cohort.

| Model | Hyper-parameter space | Best Combination of Hyperparameters |
| --- | --- | --- |
| GBC | {'learning_rate': [1, 0.5, 0.4, 0.3, 0.2, 0.1, 0.01], 'max_depth': [1, 2, 3, 4, 5, 10, 20, 30, 40], 'min_samples_leaf': [0.1, 0.3, 0.5], 'min_samples_split': [0.001, 0.1, 0.3, 0.5, 0.7, 0.9], 'n_estimators': [10, 25, 50, 100, 200, 400, 800]} | {'learning_rate': 0.01, 'max_depth': 5, 'min_samples_leaf': 0.1, 'min_samples_split': 0.1, 'n_estimators': 400} |
| LR | {'C': [0.001, 0.01, 0.1, 1, 10, 100], 'penalty':['l2'], 'solver': ['newton-cg', 'lbfgs', 'liblinear', 'sag', 'saga']} | {'C': 0.1, 'penalty': 'l2', 'solver': 'liblinear'} |
| MLP | {'activation':['tanh','relu'], 'alpha': [0.1, 0.01, 0.001, 0.0001], 'hidden_layer_sizes':[(50,),(100,)], 'solver':['sgd', 'adam'], 'learning_rate':['constant', 'adaptive']} | {'activation': 'relu', 'alpha': 0.001, 'hidden_layer_sizes': (50,), 'learning_rate': 'adaptive', 'solver': 'sgd'} |
| RF | {'max_depth': [1, 2, 3, 4, 5, 8, 10, 20, 30, 40, None], 'max_features': ['log2', 'sqrt'], 'min_samples_leaf': [1,2,4], 'min_samples_split': [2,5,10,15], 'n_estimators':[10, 25, 50, 100, 200, 400, 800]} | {'max_depth': 10, 'max_features': 'log2', 'min_samples_leaf': 2, 'min_samples_split': 10, 'n_estimators': 100} |

GBC: gradient boosting classifier; LR: logistic regression; MLP: multi-layer perceptron; RF: random forest.

**Supplementary Table 3.** Specific information on all included patients.

|  | Hemorrhagic stroke | | | Ischemic stroke | | |
| --- | --- | --- | --- | --- | --- | --- |
|  | eICU-CRD  (n=1216) | MIMIC IV  (n=921) | *P*-value | eICU-CRD  (n=954) | MIMIC IV  (n=902) | *P*-value |
| **Demographic** |  |  |  |  |  |  |
| Age | 64.47 (15.53) | 65.63 (15.04) | 0.0839 | 66.38 (14.16) | 67.75 (14.02) | 0.037 |
| Gender |  |  | 0.00104 |  |  | 0.00682 |
| Female | 656 (53.95 %) | 430 (46.69 %) |  | 502 (52.62 %) | 417 (46.23 %) |  |
| Male | 560 (46.05 %) | 491 (53.31 %) |  | 452 (47.38 %) | 485 (53.77 %) |  |
| Race |  |  | <0.001 |  |  | <0.001 |
| Asian | 17 (1.40 %) | 46 (4.99 %) |  | 6 (0.63 %) | 23 (2.55 %) |  |
| Black | 212 (17.43 %) | 90 (9.77 %) |  | 110 (11.53 %) | 89 (9.87 %) |  |
| Hispanic | 23 (1.89 %) | 40 (4.34 %) |  | 11 (1.15 %) | 25 (2.77 %) |  |
| Other/Unknown | 74 (6.09 %) | 233 (25.30 %) |  | 35 (3.67 %) | 207 (22.95 %) |  |
| White | 890 (73.19 %) | 512 (55.59 %) |  | 792 (83.02 %) | 558 (61.86 %) |  |
| Weight | 82.61 (22.55) | 80.01 (22.49) | 0.00896 | 83.88 (23.25) | 82.31 (23.30) | 0.149 |
| **Vital signs** |  |  |  |  |  |  |
| heart_rate_min | 65.04 (13.62) | 65.56 (13.63) | 0.382 | 65.13 (13.73) | 65.40 (14.65) | 0.677 |
| heart_rate_max | 102.28 (21.05) | 102.10 (19.94) | 0.839 | 99.77 (22.43) | 100.40 (21.47) | 0.544 |
| heart_rate_mean | 80.57 (14.85) | 81.29 (14.50) | 0.258 | 79.47 (15.41) | 80.18 (16.05) | 0.331 |
| sbp_min | 105.48 (20.37) | 98.36 (18.05) | <0.001 | 105.12 (20.29) | 99.43 (21.16) | <0.001 |
| sbp_max | 168.77 (26.16) | 158.97 (23.12) | <0.001 | 169.54 (27.24) | 160.17 (25.10) | <0.001 |
| sbp_mean | 134.78 (17.39) | 126.87 (14.51) | <0.001 | 135.99 (19.55) | 128.57 (19.36) | <0.001 |
| dbp_min | 52.42 (12.08) | 49.40 (11.73) | <0.001 | 52.74 (12.68) | 50.16 (13.60) | <0.001 |
| dbp_max | 98.99 (20.64) | 91.62 (19.94) | <0.001 | 101.40 (22.07) | 95.18 (21.54) | <0.001 |
| dbp_mean | 70.53 (11.02) | 66.69 (11.07) | <0.001 | 71.70 (11.66) | 67.96 (13.31) | <0.001 |
| mbp_min | 67.20 (13.21) | 62.94 (15.59) | <0.001 | 69.66 (15.72) | 64.02 (17.44) | <0.001 |
| mbp_max | 116.13 (21.38) | 111.82 (25.91) | <0.001 | 119.11 (22.64) | 113.30 (23.12) | <0.001 |
| mbp_mean | 87.50 (11.37) | 84.16 (10.21) | <0.001 | 90.60 (14.33) | 85.13 (13.57) | <0.001 |
| resp_rate_min | 12.17 (4.03) | 12.68 (3.39) | 0.00146 | 12.40 (4.26) | 12.53 (3.56) | 0.491 |
| resp_rate_max | 27.72 (8.38) | 27.29 (6.30) | 0.18 | 29.25 (8.73) | 27.73 (6.69) | <0.001 |
| resp_rate_mean | 18.42 (3.73) | 19.09 (3.62) | <0.001 | 19.14 (3.78) | 19.42 (3.85) | 0.119 |
| temperature_min | 36.25 (0.76) | 36.44 (0.78) | <0.001 | 36.31 (0.64) | 36.38 (0.77) | 0.0481 |
| temperature_max | 37.53 (0.72) | 37.63 (0.86) | 0.00894 | 37.42 (0.68) | 37.46 (0.84) | 0.259 |
| temperature_mean | 36.91 (0.52) | 37.03 (0.63) | <0.001 | 36.88 (0.48) | 36.92 (0.63) | 0.11 |
| spo2_min | 91.87 (9.06) | 92.07 (9.17) | 0.617 | 91.45 (8.04) | 91.62 (7.72) | 0.654 |
| spo2_max | 99.51 (0.98) | 99.56 (0.98) | 0.218 | 99.42 (1.25) | 99.45 (1.06) | 0.543 |
| spo2_mean | 97.28 (1.99) | 97.23 (2.43) | 0.657 | 97.10 (2.25) | 96.97 (2.18) | 0.227 |
| urineoutput | 2157.80 (1858.96) | 2003.89 (1574.12) | 0.046 | 1995.66 (1612.07) | 1735.76 (1221.80) | <0.001 |
| **Laboratory results** |  |  |  | ) | ) |  |
| aniongap_min | 11.18 (4.22) | 13.95 (3.41) | <0.001 | 10.29 (4.33) | 13.60 (3.25) | <0.001 |
| aniongap_max | 13.29 (4.81) | 16.86 (4.28) | <0.001 | 12.55 (4.87) | 16.39 (4.88) | <0.001 |
| bicarbonate_min | 23.13 (3.69) | 21.68 (4.05) | <0.001 | 23.21 (3.82) | 21.60 (4.62) | <0.001 |
| bicarbonate_max | 25.18 (3.39) | 24.04 (3.72) | <0.001 | 25.22 (3.63) | 23.97 (3.78) | <0.001 |
| creatinine_min | 1.06 (1.17) | 1.13 (1.17) | 0.186 | 1.08 (0.91) | 1.19 (1.13) | 0.0186 |
| creatinine_max | 1.24 (1.32) | 1.35 (1.37) | 0.0515 | 1.27 (1.22) | 1.41 (1.37) | 0.0245 |
| chloride_min | 101.94 (5.43) | 102.05 (5.39) | 0.63 | 102.74 (5.54) | 102.88 (5.59) | 0.582 |
| chloride_max | 105.81 (6.30) | 106.71 (6.94) | 0.00234 | 105.88 (5.45) | 106.12 (5.98) | 0.359 |
| glucose_min | 127.54 (45.72) | 127.27 (41.38) | 0.886 | 123.27 (47.74) | 123.43 (39.05) | 0.94 |
| glucose_max | 163.24 (71.70) | 173.18 (87.25) | 0.00554 | 162.78 (88.84) | 165.00 (93.34) | 0.606 |
| hematocrit_min | 35.89 (6.26) | 34.69 (6.69) | <0.001 | 35.99 (6.78) | 33.78 (7.12) | <0.001 |
| hematocrit_max | 39.15 (6.06) | 38.34 (6.07) | 0.0025 | 39.16 (6.09) | 37.18 (6.43) | <0.001 |
| hemoglobin_min | 11.96 (2.21) | 11.51 (2.32) | <0.001 | 12.01 (2.38) | 11.15 (2.49) | <0.001 |
| hemoglobin_max | 13.04 (2.16) | 12.71 (2.13) | <0.001 | 13.06 (2.21) | 12.23 (2.27) | <0.001 |
| platelet_min | 210.90 (84.84) | 197.55 (90.44) | <0.001 | 208.99 (80.19) | 201.10 (90.33) | 0.0506 |
| platelet_max | 236.21 (93.13) | 227.80 (99.81) | 0.0502 | 235.17 (89.86) | 233.31 (100.16) | 0.68 |
| potassium_min | 3.71 (0.49) | 3.80 (0.54) | <0.001 | 3.78 (0.48) | 3.92 (0.55) | <0.001 |
| potassium_max | 4.15 (0.56) | 4.47 (0.86) | <0.001 | 4.21 (0.61) | 4.46 (0.78) | <0.001 |
| inr_min | 1.11 (0.23) | 1.18 (0.29) | <0.001 | 1.14 (0.31) | 1.23 (0.36) | <0.001 |
| inr_max | 1.32 (0.75) | 1.40 (0.98) | 0.0335 | 1.24 (0.54) | 1.41 (0.78) | <0.001 |
| pt_min | 13.27 (2.63) | 12.94 (3.11) | 0.0157 | 13.52 (3.24) | 13.46 (3.91) | 0.75 |
| pt_max | 15.35 (7.78) | 15.32 (10.36) | 0.941 | 14.59 (5.61) | 15.72 (10.60) | 0.00894 |
| sodium_min | 137.55 (4.51) | 138.19 (4.81) | 0.00202 | 137.77 (4.36) | 138.59 (4.79) | <0.001 |
| sodium_max | 140.78 (5.09) | 142.47 (6.09) | <0.001 | 140.41 (4.32) | 141.44 (4.70) | <0.001 |
| bun_min | 17.73 (12.83) | 19.43 (16.29) | 0.00963 | 18.77 (12.70) | 21.61 (17.06) | <0.001 |
| bun_max | 20.81 (14.55) | 23.17 (18.57) | 0.00161 | 22.48 (15.29) | 25.37 (19.66) | <0.001 |
| wbc_min | 10.51 (10.35) | 10.55 (5.42) | 0.90 | 9.78 (4.76) | 10.25 (5.02) | 0.0421 |
| wbc_max | 12.76 (14.06) | 13.58 (7.60) | 0.0881 | 11.94 (6.12) | 13.38 (7.08) | <0.001 |
| calcium_min | 8.49 (0.88) | 8.44 (0.83) | 0.133 | 8.41 (0.94) | 8.38 (0.87) | 0.455 |
| calcium_max | 8.99 (0.68) | 8.88 (0.74) | 0.0016 | 8.95 (0.68) | 8.81 (0.88) | <0.001 |
| **Severity scores on admission** |  |  |  |  |  |  |
| Charlson comorbidity index | 3.37 (2.48) | 5.29 (2.77) | <0.001 | 3.76 (2.35) | 6.11 (2.90) | <0.001 |
| GCS | 11.43 (4.37) | 12.40 (3.84) | <0.001 | 12.34 (3.61) | 13.03 (3.36) | <0.001 |
| APSIII | 46.29 (26.13) | 43.49 (23.86) | 0.0113 | 43.15 (24.15) | 44.07 (23.93) | 0.422 |
| SOFA | 3.57 (2.76) | 4.21 (3.65) | <0.001 | 3.28 (2.88) | 4.30 (3.81) | <0.001 |
| **First day treatment** |  |  |  |  |  |  |
| Vasopressor | 62 (5.10 %) | 186 (20.20 %) | <0.001 | 73 (7.65 %) | 197 (21.84 %) | <0.001 |
| Renal replacement therapy | 13 (1.07 %) | 24 (2.61 %) | 0.0114 | 13 (1.36 %) | 23 (2.55 %) | 0.092 |
| Mechanical ventilation | 445 (36.60 %) | 421 (45.71 %) | <0.001 | 277 (29.04 %) | 303 (33.59 %) | 0.191 |
| Hospital length of stay, day | 8.78 (8.74) | 8.57 (9.72) | 0.622 | 7.50 (11.59) | 9.30 (11.46) | <0.001 |
| ICU length of stay, day | 5.86 (6.39) | 7.61 (8.06) | <0.001 | 4.44 (5.72) | 7.90 (9.86) | <0.001 |
| **Neurological outcome** |  |  |  |  |  |  |
| Favorable | 809 (66.53 %) | 454 (49.29 %) | <0.001 | 711 (74.53 %) | 540 (59.87 %) | <0.001 |
| Unfavorable | 407 (33.47 %) | 467 (50.71 %) |  | 243 (25.47 %) | 362 (40.13 %) |  |

Data are n (%) or mean (SD). Two-tailed Student’s t-tests or Mann-Whitney U-test for continuous variables, Chi-squared or Fisher’s exact test for categorical variables. SBP: systolic blood pressure; DBP: diastolic blood pressure; MBP: mean blood pressure; SpO2: oxygen saturation; INR: international normalized ratio; PT: prothrombin time; BUN: blood urea nitrogen; WBC: white blood cell; GCS: Glasgow Coma Scale; APSIII: Acute Physiology Score III; SOFA: Sequential Organ Failure Assessment.

**Supplementary Table 4.** Specific information on hemorrhagic and ischemic stroke patients across training and testing sets.

|  | Hemorrhagic stroke | |  | Ischemic stroke | |  |
| --- | --- | --- | --- | --- | --- | --- |
|  | Training set (n=851) | Testing set (n=365) | *P*-value | Training set (n=667) | Testing set (n=287) | *P*-value |
| **Demographic** |  |  |  |  |  |  |
| Age | 64.64 (15.35) | 64.09 (15.96) | 0.577 | 65.67 (14.21) | 68.02 (13.92) | 0.0178 |
| Gender |  |  | 0.669 |  |  | 0.83 |
| Female | 463 (54.41 %) | 193 (52.88 %) |  | 353 (52.92 %) | 149 (51.92 %) |  |
| Male | 388 (45.59 %) | 172 (47.12 %) |  | 314 (47.08 %) | 138 (48.08 %) |  |
| Race |  |  | 0.882 |  |  | 0.965 |
| Asian | 12 (1.41 %) | 5 (1.37 %) |  | 5 (0.75 %) | 1 (0.35 %) |  |
| Black | 150 (17.63 %) | 62 (16.99 %) |  | 77 (11.54 %) | 33 (11.50 %) |  |
| Hispanic | 18 (2.12 %) | 5 (1.37 %) |  | 7 (1.05 %) | 4 (1.39 %) |  |
| Other/Unknown | 49 (5.76 %) | 25 (6.85 %) |  | 24 (3.60 %) | 11 (3.83 %) |  |
| White | 622 (73.09 %) | 268 (73.42 %) |  | 554 (83.06 %) | 238 (82.93 %) |  |
| Weight | 82.61 (22.44) | 82.60 (22.82) | 0.999 | 83.26 (23.16) | 85.33 (23.42) | 0.213 |
| **Vital signs** |  |  |  |  |  |  |
| heart_rate_min | 64.71 (13.09) | 65.80 (14.78) | 0.225 | 65.72 (13.92) | 63.74 (13.20) | 0.0386 |
| heart_rate_max | 102.30 (20.72) | 102.24 (21.84) | 0.965 | 100.24 (22.44) | 98.69 (22.43) | 0.329 |
| heart_rate_mean | 80.48 (14.30) | 80.76 (16.10) | 0.776 | 80.19 (15.68) | 77.81 (14.63) | 0.0255 |
| sbp_min | 105.31 (20.66) | 105.90 (19.71) | 0.639 | 105.22 (20.92) | 104.88 (18.75) | 0.809 |
| sbp_max | 170.04 (26.71) | 165.81 (24.61) | 0.00807 | 169.67 (27.59) | 169.26 (26.46) | 0.833 |
| sbp_mean | 135.14 (17.31) | 133.95 (17.57) | 0.283 | 135.98 (19.76) | 136.04 (19.10) | 0.967 |
| dbp_min | 52.22 (12.04) | 52.88 (12.17) | 0.388 | 53.14 (13.10) | 51.80 (11.62) | 0.121 |
| dbp_max | 99.58 (21.10) | 97.61 (19.46) | 0.117 | 101.96 (22.59) | 100.10 (20.79) | 0.221 |
| dbp_mean | 70.73 (10.90) | 70.07 (11.30) | 0.347 | 72.05 (11.85) | 70.89 (11.20) | 0.155 |
| mbp_min | 67.23 (13.17) | 67.13 (13.31) | 0.90 | 70.01 (16.48) | 68.85 (13.78) | 0.268 |
| mbp_max | 116.54 (21.63) | 115.17 (20.78) | 0.301 | 120.01 (22.81) | 117.01 (22.11) | 0.0602 |
| mbp_mean | 87.71 (11.16) | 87.02 (11.84) | 0.348 | 91.07 (14.72) | 89.50 (13.35) | 0.109 |
| resp_rate_min | 12.08 (3.91) | 12.38 (4.30) | 0.251 | 12.35 (4.20) | 12.52 (4.39) | 0.59 |
| resp_rate_max | 27.79 (8.55) | 27.55 (8.00) | 0.635 | 29.09 (8.60) | 29.61 (9.06) | 0.418 |
| resp_rate_mean | 18.34 (3.68) | 18.59 (3.84) | 0.312 | 19.00 (3.67) | 19.46 (4.02) | 0.103 |
| temperature_min | 36.25 (0.77) | 36.26 (0.75) | 0.818 | 36.32 (0.63) | 36.30 (0.66) | 0.728 |
| temperature_max | 37.53 (0.69) | 37.55 (0.79) | 0.595 | 37.41 (0.68) | 37.44 (0.69) | 0.524 |
| temperature_mean | 36.91 (0.50) | 36.92 (0.55) | 0.717 | 36.87 (0.46) | 36.88 (0.53) | 0.76 |
| spo2_min | 91.94 (8.88) | 91.71 (9.46) | 0.696 | 91.31 (8.70) | 91.77 (6.25) | 0.378 |
| spo2_max | 99.50 (0.96) | 99.51 (1.03) | 0.867 | 99.47 (0.96) | 99.29 (1.73) | 0.103 |
| spo2_mean | 97.27 (2.04) | 97.29 (1.89) | 0.891 | 97.12 (2.16) | 97.04 (2.45) | 0.658 |
| urineoutput | 2141.78 (1955.82) | 2194.82 (1615.19) | 0.64 | 2024.42 (1613.65) | 1926.95 (1609.40) | 0.42 |
| **Laboratory results** | ) | ) |  | ) | ) |  |
| aniongap_min | 11.12 (4.17) | 11.30 (4.32) | 0.548 | 10.30 (4.34) | 10.29 (4.30) | 0.973 |
| aniongap_max | 13.32 (4.76) | 13.21 (4.91) | 0.754 | 12.53 (4.87) | 12.58 (4.88) | 0.888 |
| bicarbonate_min | 23.11 (3.66) | 23.19 (3.77) | 0.738 | 23.12 (3.93) | 23.40 (3.55) | 0.313 |
| bicarbonate_max | 25.23 (3.32) | 25.06 (3.56) | 0.441 | 25.15 (3.72) | 25.39 (3.41) | 0.354 |
| creatinine_min | 1.04 (1.03) | 1.12 (1.44) | 0.348 | 1.06 (0.88) | 1.12 (0.97) | 0.379 |
| creatinine_max | 1.22 (1.21) | 1.28 (1.54) | 0.514 | 1.26 (1.24) | 1.30 (1.16) | 0.634 |
| chloride_min | 101.91 (5.21) | 101.99 (5.92) | 0.827 | 102.86 (5.33) | 102.45 (5.99) | 0.322 |
| chloride_max | 105.90 (6.15) | 105.62 (6.64) | 0.496 | 106.03 (5.39) | 105.53 (5.59) | 0.214 |
| glucose_min | 128.72 (47.37) | 124.84 (41.63) | 0.16 | 123.45 (47.08) | 122.86 (49.32) | 0.867 |
| glucose_max | 164.57 (72.99) | 160.17 (68.65) | 0.321 | 163.09 (90.61) | 162.06 (84.74) | 0.871 |
| hematocrit_min | 35.95 (6.25) | 35.75 (6.30) | 0.613 | 35.95 (6.81) | 36.09 (6.71) | 0.779 |
| hematocrit_max | 39.35 (5.95) | 38.70 (6.29) | 0.104 | 39.13 (5.99) | 39.20 (6.32) | 0.878 |
| hemoglobin_min | 11.98 (2.21) | 11.91 (2.19) | 0.605 | 12.00 (2.40) | 12.04 (2.35) | 0.815 |
| hemoglobin_max | 13.10 (2.13) | 12.89 (2.20) | 0.151 | 13.06 (2.18) | 13.06 (2.28) | 0.991 |
| platelet_min | 213.01 (83.26) | 206.01 (88.32) | 0.206 | 208.57 (82.23) | 209.96 (75.36) | 0.805 |
| platelet_max | 239.50 (92.35) | 228.63 (94.61) | 0.07 | 235.04 (92.77) | 235.47 (82.81) | 0.945 |
| potassium_min | 3.70 (0.48) | 3.74 (0.51) | 0.279 | 3.79 (0.48) | 3.77 (0.49) | 0.662 |
| potassium_max | 4.15 (0.58) | 4.15 (0.51) | 0.973 | 4.25 (0.64) | 4.14 (0.52) | 0.00955 |
| inr_min | 1.11 (0.24) | 1.11 (0.21) | 0.963 | 1.15 (0.33) | 1.12 (0.26) | 0.192 |
| inr_max | 1.33 (0.80) | 1.28 (0.61) | 0.301 | 1.27 (0.60) | 1.18 (0.34) | 0.00897 |
| pt_min | 13.24 (2.68) | 13.34 (2.50) | 0.582 | 13.58 (3.42) | 13.39 (2.78) | 0.479 |
| pt_max | 15.52 (8.50) | 14.98 (5.86) | 0.269 | 14.86 (6.23) | 13.99 (3.77) | 0.0352 |
| sodium_min | 137.53 (4.27) | 137.59 (5.02) | 0.858 | 137.82 (4.21) | 137.64 (4.69) | 0.59 |
| sodium_max | 140.88 (5.02) | 140.54 (5.26) | 0.297 | 140.56 (4.28) | 140.05 (4.40) | 0.111 |
| bun_min | 17.30 (11.92) | 18.72 (14.68) | 0.107 | 18.48 (12.14) | 19.46 (13.90) | 0.314 |
| bun_max | 20.36 (13.42) | 21.83 (16.85) | 0.145 | 22.16 (15.12) | 23.23 (15.67) | 0.341 |
| wbc_min | 10.72 (12.00) | 10.00 (4.56) | 0.134 | 9.82 (4.98) | 9.69 (4.22) | 0.685 |
| wbc_max | 13.07 (16.39) | 12.03 (5.66) | 0.109 | 11.93 (6.28) | 11.97 (5.74) | 0.921 |
| calcium_min | 8.48 (0.91) | 8.52 (0.81) | 0.50 | 8.40 (0.98) | 8.45 (0.85) | 0.389 |
| calcium_max | 9.00 (0.66) | 8.96 (0.71) | 0.466 | 8.95 (0.69) | 8.94 (0.67) | 0.934 |
| **Severity scores on admission** |  |  |  |  |  |  |
| Charlson comorbidity index | 3.43 (2.51) | 3.24 (2.42) | 0.227 | 3.69 (2.36) | 3.93 (2.35) | 0.159 |
| GCS | 11.44 (4.40) | 11.41 (4.30) | 0.921 | 12.30 (3.63) | 12.45 (3.56) | 0.553 |
| APSIII | 46.11 (26.33) | 46.72 (25.69) | 0.713 | 43.42 (24.41) | 42.53 (23.57) | 0.611 |
| SOFA | 3.55 (2.75) | 3.62 (2.79) | 0.692 | 3.20 (2.80) | 3.45 (3.05) | 0.247 |
| **First day treatment** |  |  |  |  |  |  |
| Vasopressor | 45 (5.29 %) | 17 (4.66 %) | 0.752 | 53 (7.95 %) | 20 (6.97 %) | 0.698 |
| Renal replacement therapy | 9 (1.06 %) | 4 (1.10 %) | 1.00 | 8 (1.20 %) | 5 (1.74 %) | 0.546 |
| Mechanical ventilation | 305 (35.84 %) | 140 (38.36 %) | 0.469 | 196 (29.39 %) | 81 (28.22 %) | 0.783 |
| Hospital length of stay, day | 8.70 (8.49) | 8.94 (9.31) | 0.678 | 7.31 (7.78) | 7.93 (17.51) | 0.562 |
| ICU length of stay, day | 5.91 (6.37) | 5.75 (6.45) | 0.687 | 4.51 (5.94) | 4.28 (5.16) | 0.545 |

Data are n (%) or mean (SD). Two-tailed Student’s t-tests or Mann-Whitney U-test for continuous variables, Chi-squared or Fisher’s exact test for categorical variables. SBP: systolic blood pressure; DBP: diastolic blood pressure; MBP: mean blood pressure; SpO2: oxygen saturation; INR: international normalized ratio; PT: prothrombin time; BUN: blood urea nitrogen; WBC: white blood cell; GCS: Glasgow Coma Scale; APSIII: Acute Physiology Score III; SOFA: Sequential Organ Failure Assessment.

**Supplementary Table 5.** Model performance summary for MIMIC external validation in hemorrhagic stroke cohort.

| Model | Accuracy | PPV | NPV | Sensitivity | Specificity | F1 | AUC |
| --- | --- | --- | --- | --- | --- | --- | --- |
| GBC | 0.772 | 0.767 | 0.777 | 0.790 | 0.753 | 0.778 | 0.826 |
| LR | 0.777 | 0.768 | 0.788 | 0.803 | 0.751 | 0.785 | 0.836 |
| MLP | 0.748 | 0.782 | 0.720 | 0.698 | 0.800 | 0.738 | 0.812 |
| RF | 0.824 | 0.763 | 0.927 | 0.946 | 0.698 | 0.845 | 0.881 |

GBC: gradient boosting classifier; LR: logistic regression; MLP: multi-layer perceptron; RF: random forest; PPV: positive predictive value; NPV: negative predictive value; F1: F-measure; AUC: area under curve.

**Supplementary** **Table 6.** Model performance summary for MIMIC external validation in ischemic stroke cohort.

| Model | Accuracy | PPV | NPV | Sensitivity | Specificity | F1 | AUC |
| --- | --- | --- | --- | --- | --- | --- | --- |
| GBC | 0.761 | 0.647 | 0.901 | 0.890 | 0.674 | 0.749 | 0.841 |
| LR | 0.766 | 0.675 | 0.849 | 0.804 | 0.741 | 0.734 | 0.833 |
| MLP | 0.733 | 0.632 | 0.837 | 0.801 | 0.687 | 0.706 | 0.807 |
| RF | 0.787 | 0.673 | 0.924 | 0.914 | 0.702 | 0.775 | 0.856 |

GBC: gradient boosting classifier; LR: logistic regression; MLP: multi-layer perceptron; RF: random forest; PPV: positive predictive value; NPV: negative predictive value; F1: F-measure; AUC: area under curve.
